# The Impact of Nitrogen and Phosphorus Interaction on Growth, Nutrient Absorption, and Signal Regulation in Woody Plants

**DOI:** 10.3390/biology14050490

**Published:** 2025-04-30

**Authors:** Xiaan Tang, Yi Zhang, Panpan Meng, Yingke Yuan, Changhao Li, Xiaotan Zhi, Chunyan Wang

**Affiliations:** College of Forestry, Northwest A&F University, Yangling, Xianyang 712100, China; 15078371592@163.com (X.T.); zhangyi.1978@163.com (Y.Z.); 18391861476@163.com (P.M.); yuanyingk@126.com (Y.Y.); 13017532728@163.com (C.L.); 15131648016@163.com (X.Z.)

**Keywords:** nitrogen–phosphorus interactions, woody plants, nutrient uptake, root system architecture, transcription factors

## Abstract

This article methodically reveals how, in woody plants (poplar), the interaction between N and P coordinates root structure and nutrient absorption through a complex hormone signaling network. This study bridges a significant gap in our knowledge of nutrient interaction networks. The results demonstrate that NO_3_^−^ significantly enhances the gene expression and enzymatic activity of organic acid synthases (MDH, PEPC) and APs. Furthermore, it synergizes with IAA/ABA signals to refine root structure, enhancing the surface area for P absorption. In low Pi availability environments, NO_3_^−^ further promotes P recycling by simultaneously boosting the levels of Pi transport proteins (notably, the PHO family), facilitating myo-inositol phosphate metabolism (via IMP3/ITPK1-mediated PP-InsPs degradation), and augmenting IAA/SA signals. Pi induces the activity of N assimilation enzymes (GS/GOGAT/GDH), facilitating nitrogen metabolism. However, in the absence of N, it leads to a metabolic imbalance characterized by high enzymatic activity but low efficiency. Alternatively, adequate N availability allows Pi to improve root robustness and N assimilation efficiency, mediated by IAA/GA accumulation and ABA signaling (e.g., *SNRK2*/*ABF*). We propose the existence of an intricate network in poplar, orchestrated by transcriptional cascades, metabolic regulation, and hormonal synergism. Key modules such as *SPX*-*PHR*, *NLA*, *HHO2*, and *MYB59* are likely central to this network’s function. These findings offer a foundational framework for the development of molecular breeding and precise fertilization strategies, enhancing the efficient use of N and P in forestry.

## Simple Summary

This study elucidates the intrinsic mechanisms through which poplars optimize root architecture and enhance nutrient uptake efficiency via the synergistic action of N and P. These elements, pivotal for plant growth, have long been recognized as having a synergistic mechanism that has remained largely unexplored in woody plants. The research demonstrates that N amplifies the activity of crucial enzymes and proteins related to P absorption, facilitating root expansion to increase P capture. In low Pi availability environments, N catalyzes recycling processes for reusing P, whereas P aids in N assimilation within the plant. Nonetheless, the absence of a balanced nutrient supply impedes plant growth efficiency. Further, this study unveils the role of natural plant hormones and genetic pathways in orchestrating these processes. These findings expose a collaborative interplay among nutrients, genes, and hormones that is crucial for root development and nutrient optimization. Comprehending this natural system paves the way for formulating advanced strategies in tree breeding or forest fertilization, minimizing waste and ecological damage. Accordingly, this research contributes to sustainable forestry by promoting the growth of healthier trees with minimized resource utilization, an essential endeavor in the fight against climate change and ecosystem preservation.

## 1. Introduction

N and P are vital macronutrients for plant growth and development [1]. Plants mainly take up and utilize phosphorus in the form of inorganic phosphate (Pi). However, Pi’s low solubility and mobility in soil often result in minimal plant availability [2]. N is essential for plants as it is a key element in proteins, nucleic acids, chlorophylls, and various secondary metabolites. Nitrate (NO_3_^−^) is the primary N source for most terrestrial plants, although its availability can substantially vary across locations and over time [3].

To mitigate N and P deficiencies in their environment, plants have evolved a suite of adaptive strategies aimed at improving their capacity to absorb and utilize these nutrients [4,5]. Plant roots are directly responsible for the exploration and absorption of nitrogen and phosphorus elements in the soil. Therefore, plants typically adjust their root system structure to adapt to different N and P nutritional conditions. Low N mainly stimulated root elongation [6], but P deficiency increased root branching [7]. The combined application of nitrogen and phosphorus enhanced root surface area, root length, and root bud quality [8]. The addition of N and P influences their absorption and each other’s uptake by impacting root development. Environmental factors modulate root architecture through alterations in plant hormone dynamics and sensitivity [9]. Auxin, a pivotal hormone for root growth and development, interacts with N and P availability to regulate these processes. N and P influence root development by altering hormone biosynthesis, signal transduction, and polar transport. The research of Tian et al. [10] demonstrated that NO_3_^−^ supply reduced indole-3-acetic acid (IAA) levels in roots; however, the application of exogenous IAA alleviated the inhibitory impact of high nitrogen on primary root elongation. Similarly, P scarcity led to enhanced lateral root formation and inhibited primary root growth when plants were treated with external auxin [11]. Abscisic acid (ABA) is considered a growth inhibitor [12]. Elevated nitrate levels promote ABA accumulation in the root endoderm and meristematic zones, aiding in meristem maintenance [13]. Moreover, phosphate availability modulates root development through interactions with ABA signaling [14]. Low N conditions were found to activate gibberellin (GA) and jasmonic acid (JA) signaling, altering the hormonal signal transduction network in plants [15]. Moreover, P stress decreases GA bioactivity in *Arabidopsis thaliana*, affecting root architecture and suggesting that optimal GA levels are vital for root growth and development [16]. Research has also found that salicylic acid (SA) enhances phosphorus uptake by modulating the expression of phosphorus transporter proteins [17]. The restructuring of the root architecture, driven by the nutritional environment, essentially establishes the physical interface for N and P absorption, while the synergistic regulatory mechanisms at the molecular level further optimize the coupled utilization efficiency of nutrients.

The synergistic regulation of N and P at the molecular level plays a critical role in maintaining the balance of N and P nutrition in plants [18]. This interaction includes the N-regulated P response and the P-regulated N response. The former has been extensively studied; for instance, phosphate availability has been shown to significantly influence the expression of nitrate response genes in both *Arabidopsis thaliana* and *Oryza sativa* [19,20,21]. AtNRT1.5, a nitrate transporter in *Arabidopsis thaliana*, is instrumental in root-to-shoot NO_3_^−^ transport and regulates the plant’s response to Pi scarcity [22]. Furthermore, N supplementation has been reported to trigger Pi starvation responses, bolstering plant resistance to P stress [21]. Conversely, the P-regulated N response has received less attention. Transcriptome analyses in *Arabidopsis* have revealed that several Pi/N response genes, such as *NIA1*, *NIR*, and *CHL1/NRT1.1*, are suppressed as soon as 24 h following the initiation of P deprivation [23], highlighting a close association between the phosphate starvation response (PSR) and primary nitrate response (PNR) pathways. Although research on certain model plants has revealed the core regulatory nodes of N and P interactions, the unique secondary growth patterns and long-term nutrient storage strategies of woody plants may reshape their synergistic regulation network, necessitating further research and clarification. Based on the evidence, we hypothesize that the availability of N and P influences will differentially regulate the root morphology of woody plants, the efficiency of nutrient absorption, and the expression of genes related to the absorption and metabolism of each other. This is primarily achieved through the interaction between hormonal signaling pathways (such as IAA, ABA, and GA) and nutrition response genes (such as *NRT1.5*, *NIA1*, PSR/PNR related genes), which, in turn, regulate the structure of the root system and the expression of transport proteins, affecting their absorption and utilization.

As a model species for woody plant research, *Populus* spp. has had its genome widely deciphered [24]. This species has a wide distribution in the temperate and northern forest regions of the Northern Hemisphere, where in its native environment, the supply of nitrogen and phosphorus often exhibits significant imbalances, particularly with a marked deficiency of soil Pi [25]. To sustain the demands for rapid growth, large amounts of nitrogen and phosphorus fertilizers are often applied in the management of poplar plantations to overcome soil nutrient limitations. However, this practice not only leads to low fertilizer use efficiency but also results in a series of environmental issues. Therefore, it is critically important for both theory and practice to conduct an in-depth analysis of the molecular mechanisms underlying the synergistic absorption and utilization of N and P in poplars. Coordinating the acquisition of N and P nutrients is essential for developing targeted fertilization strategies, enhancing nutrient use efficiency, and reducing ecological and environmental risks. This study investigates the interaction between N and P nutrition in *Populus alba* × *Populus glandulosa* (84 K poplar) roots under different N-P treatments, focusing on (1) the effects on root morphology and physiology, and (2) the underlying mechanisms of these interactions.

## 2. Materials and Methods

### 2.1. Materials

Initially, 84 K poplar saplings derived from micropropagation were grown for one month in an artificial climate chamber. These saplings were then transferred to columnar flowerpots containing fine sand, with each pot containing one seedling that was 20 cm deep and 10 cm in diameter. Following transplantation, they were further cultivated and received treatments in a greenhouse (natural light; day/night temperature: 28/20 °C; relative humidity: 75%). The saplings received 50 mL of Hoagland solution for irrigation every two days. After cultivation for 1 week, the seedlings were transferred to a new basin filled with 0.8 kg of clean fine sand, and 50 mL of pure water was irrigated every 2 days. Plants with comparable height (~15 cm) and growth performance were chosen for nutritional treatments after one week of treatment. Each plant was gradually irrigated with 50 mL of a modified Hoagland solution containing 2 mM MgSO_4_·7H_2_O, 1 mM KCl, 0.5 mM CaSO_4_·2H_2_O, 45 μM H₃BO₃, 10 μM MnCl_2_·4H_2_O, 0.8 μM ZnSO4·7H_2_O, 0.3 μM CuSO₄·5H_2_O, 0.4 μM Na_2_MoO_4_·2H_2_O, 20 μM FeSO_4_·7H_2_O, and 20 μM EDTA-2Na, with varying concentrations of KNO_3_ (N) and KH_2_PO_4_ (P) as follows: 0 mM N and 0 mM P (−N, −P), 30 mM N and 0 mM P (+N, −P), 0 mM N and 1.5 mM P (−N, +P), and 30 mM N and 1.5 mM P (+N, +P). Nutritional treatments were administered for 60 days to gather adequate plant material for physiological analyses. The experiment comprised 60 seedlings in total (2N treatments × 2P treatments × 15 repeats).

### 2.2. Harvesting

The roots were harvested and thoroughly washed with the designated nutrient solution after 60 days of treatment. Utilizing the method outlined by Luo et al. [25], root samples were scanned in five replicates using the WinRHIZO system (WinRHIZO 2012b, Regent Instruments Canada, Inc., Montreal, QC, Canada). The samples were frozen, ground with liquid nitrogen, and stored at −80 °C. Dry weight was measured by oven-drying at 85 °C until a stable weight was reached.

### 2.3. Assessment of P and N Concentrations

Five biological replicates from each treatment were selected for assessment of P and N concentrations, each subject to three technical duplications. The protocol by Wang et al. [26] was followed, wherein plant dry powder was digested using sulfuric acid and hydrogen peroxide. The N and P concentrations in the digest were analyzed at 660 nm and 700 nm, respectively, using a Continuous-Flow Analyzer (AA3, Bran-Luebbe, Hamburg, Germany), as per the method by Gan et al. [27]. Phosphorus utilization efficiencies (PUEs) and nitrogen utilization efficiencies (NUEs) were calculated using the formula: efficiency = biomass/nutrient uptake, following the guidelines of Gan et al. [27].

### 2.4. Determination of Enzymatic Activities

Five biological replicates from each treatment were selected for enzyme activity assays, each subject to three technical duplications. Phosphatase (APs, EC 3.1.3.2) activity in root samples was evaluated according to the method described by Lei et al. [28]. A 50 mg finely powdered sample was extracted using 1.8 mL of 0.04 M sodium acetate buffer at pH 6.5. A 100 μL extract was mixed with 600 μL of 15 mM sodium 4-nitrophenyl phosphate and 1.3 mL of 40 mM sodium acetate, followed by a 30 min incubation at 37 °C in the dark. Absorbance was measured at 412 nm by spectrophotometry. The activities of phosphoenolpyruvate carboxylase (PEPC, EC 4.1.1.31) and malate dehydrogenase (MDH, EC 1.1.1.37) were assessed following the procedures outlined by Gajewska et al. [29] and Lü et al. [30], which involved the extraction of 100 mg from a frozen powdered sample. The PEPC assay involved combining 100 μL of extract with 900 μL of a reaction mixture comprising 25 mM Tris-HCl (pH 8.0), 5 mM MgCl_2_·6H_2_O, 1 mM DTT, 2 mM KHCO₃, 0.1 mM NADH, 3U MDH, and 2.5 mM phosphoenolpyruvate, followed by incubation at 37 °C for 5 min. For MDH, a 900 μL reaction mixture containing 25 mM Tris-HCl (pH 8.0), 0.1 mM NADH, and 0.15 mM oxaloacetic acid was preheated at 37 °C for 5 min prior to initiating the reaction with 100 μL of extract. The decrease in absorbance at 340 nm was recorded over 5 min. Moreover, the enzymatic activities of glutamine synthetase (GS, EC 6.3.1.2), nitrate reductase (NR, EC 1.7.99.4), glutamate synthase (GOGAT, EC 1.4.7.1), and glutamate dehydrogenase (GDH, EC 1.4.1.2) in poplar roots were thoroughly analyzed using the method outlined by Luo et al. [25].

### 2.5. Levels of Malic and Citric Acids

Five biological replicates from each treatment were selected to measure the concentrations of malic acid and citric acid, each subject to three technical duplications. Malic and citric acid concentrations in root samples were measured following Lü et al. [30] and Dong et al.’s [31] protocols. Initially, samples were homogenized in 80% ethanol and intermittently oscillated at 80 °C for 20 min. Following cooling, the homogenate was centrifuged to extract the supernatant, and subsequently dried under N_2_. The dry residues were dissolved in ultrapure water and analyzed using High-Performance Liquid Chromatography (HPLC; Agilent 1260 Infinity, Agilent Technologies, Santa Clara, CA, USA). A C18 column (5 µm, 250 × 4.6 mm, Agilent) facilitated chromatographic separation with a mobile phase comprising 0.02 M KH_2_PO_4_ (pH 2.8) and 3% methanol. The column was operated at 17 °C with a flow rate of 0.5 mL/min, and detection was performed at 210 nm.

### 2.6. Levels of Phytohormone Concentrations

Five biological replicates from each treatment were selected for the determination of hormone concentration levels, each subject to three technical duplications. Plant hormones, such as ABA, GA_3_, IAA, SA, and JA, were extracted from fresh root powder by homogenization in 80% methanol with 200 mg·L^−1^ butylated hydroxytoluene and 500 mg·L^−1^ citric acid monohydrate. After shaking and centrifugation, the supernatant was collected, dried under N_2_, and resuspended in 80% methanol. The solution was analyzed with a Shimadzu LC-20AT HPLC system paired with an API 2000™ electrospray tandem mass spectrometer. The chromatographic analysis was performed using an Eclipse XDB-C18 column (250 × 4.6 mm, 5 μm) and plant hormone standards from Sigma Chemical Co. (St. Louis, MO, USA) to develop quantitative standard curves [32].

### 2.7. Measurement of Carbohydrate Levels

Five biological replicates from each treatment were selected for the determination of carbohydrate levels, each subject to three technical duplications. The levels of soluble sugars and starch in root samples were quantified using the anthrone method described by He et al. [33]. Briefly, 100 mg of fresh fine root powder was homogenized in 4 mL of 80% ethanol and incubated at 75 °C for 35 min with intermittent shaking. The homogenate was centrifuged at 8500× *g* for 15 min at 25 °C, and the supernatant was collected post-cooling. As previously mentioned, the precipitate was re-extracted, and the supernatant from this process was combined with the initial one. A total of 2 mL of anthrone reagent was added to the combined supernatants and heated in boiling water for 15 min. After cooling to room temperature, the absorbance of the solution was measured spectrophotometrically at 620 nm. A standard curve was generated from a range of diluted glucose solutions.

### 2.8. Ribonucleic Acid (RNA) Isolation and Sequencing

Root tissues from four treatment groups (three biological replicates per treatment) underwent total RNA extraction using TRIzol^®^ Reagent (Thermo Fisher Scientific, Waltham, MA, USA), in accordance with the manufacturer’s guidelines. The RNA quality was assessed with a 5300 Bioanalyser (Agilent), and quantification was performed using the ND-2000 (NanoDrop Technologies (Thermo Fisher Scientific, Wilmington, DE, USA). Only high-quality RNA samples (OD260/280 = 1.8~2.2, OD260/230 ≥ 2.0, RQN ≥ 6.5, and 28S:18S ≥ 1.0; quantity > 1 μg) were utilized for sequencing library construction. RNA purification, reverse transcription, library construction, and sequencing were conducted at Shanghai Majorbio Bio-pharm Biotechnology Co., Ltd. (Shanghai, China) following the manufacturer’s instructions. The root tissue RNA-seq transcriptome library was prepared with 1 µg of total RNA using the Illumina^®^ Stranded mRNA Prep, Ligation kit (San Diego, CA, USA). Messenger RNA was isolated via the polyA selection method using oligo (dT) beads, followed by fragmentation. Double-stranded cDNA was synthesized using a SuperScript cDNA synthesis kit (Thermo Fisher Scientific, Carlsbad, CA, USA) with random hexamer primers, followed by end-repair, phosphorylation, and adapter ligation, according to the library construction protocol. cDNA libraries with 300 bp target fragments were selected using 2% Low Range Ultra Agarose and amplified through 15 PCR cycles with Phusion DNA polymerase (NEB (New England Biolabs, Ipswich, MA, USA). Following quantification with Qubit 4.0, sequencing was performed on the NovaSeq X Plus or DNBSEQ-T7 platform using the respective kits.

The raw paired-end reads were trimmed and quality controlled by fastp [34] with default parameters. The specific steps are as follows: First, adapter sequences were removed, and reads without inserts due to adapter self-ligation were filtered out. Subsequently, the average quality value was calculated using a 4 bp sliding window method. Bases at the 5′ end with an average quality score below 20 were trimmed iteratively, while bases at the 3′ end with a quality score less than 3 were truncated. Furthermore, low-quality sequences containing more than 5 ambiguous bases (N) were filtered out. Finally, high-confidence clean reads with a length ≥ 30 bp after the above processing were retained for subsequent analysis. Then, clean reads were separately aligned to the *P. alba* reference genome (https://ftp.cngb.org/pub/CNSA/data1/CNP0000339/CNS0047055/CNA0003521/, accessed on 1 June 2024) with orientation mode using HISAT2 with default parameters [35]. The mapped reads of each sample were assembled by StringTie [36] using a reference-based approach to obtain the number of fragments per kilobase of exon per million mapped reads (FPKMs) for genes.

### 2.9. RNA Sequencing Analysis, Differential Expression Analysis, and Functional Enrichment

To identify DEGs (differentially expressed genes) between two different samples, the expression level of each transcript was calculated according to the FPKM method. For transcriptome profiling, raw read counts were quantified using RSEM [37] with Bowtie2 alignment under default parameters (end-to-end mode, ≤2 mismatches allowed per read). Differential expression analysis was performed in DESeq2 [38], applying variance stabilizing transformation with normalized library sizes. Genes with average counts < 10 across samples were filtered prior to statistical testing. We defined differentially expressed genes (DEGs) as those meeting both thresholds: (i) false discovery rate (FDR)-adjusted *p*-value (Benjamini–Hochberg procedure) < 0.05, and (ii) absolute |log2 (fold change)| > 1.

Additionally, functional enrichment analysis, encompassing both Gene Ontology (GO) and Kyoto Encyclopedia of Genes and Genomes (KEGG), was conducted. This analysis sought to identify significant enrichment of DEGs in GO terms and metabolic pathways, applying a Bonferroni-corrected *p*-value < 0.05 against the whole-transcriptome background. GO functional enrichment and KEGG pathway analyses were performed using Goatools (v1.3.0) and a Python (v3.8.10)script.

### 2.10. Functional Classification and Comparative Analysis of Transcription Factors (TFs)

To identify TFs in DEGs, we referred to the following online databases: NCBI (www.ncbi.nlm.nih.gov, accessed on 10 June 2024), PopGenie (https://popgenie.org/, accessed on 10 June 2024), Pfam (https://www.ebi.ac.uk/interpro/, accessed on 10 June 2024), EggNOG (https://eggnogdb.embl.de/, accessed on 10 June 2024), Swiss-Prot (https://web.expasy.org/docs/swiss-prot_guideline.html, accessed on 10 June 2024), and Plant TFDB (http://planttfdb.gao-lab.org/, accessed on 10 June 2024). Transcription factors were categorized into functional groups using MapMan (v3.6.0), with *P. alba* serving as the reference genome from PopGenie (https://popgenie.org/, accessed on 11 June 2024) and Phytozome (https://phytozome-next.jgi.doe.gov/, accessed on 11 June 2024).

### 2.11. Quantitative Real-Time PCR (qRT-PCR) Validation

To validate the RNA-seq results, the expression levels of 12 selected genes were assessed through qRT-PCR. Total RNA was extracted following the procedure described by Gan et al. [27]; the Poplar_84k_genome_v1.0 database was used to design specific primers. *Actin 2/7a* gene was used as the housekeeping gene for normalization, with amplification systems and protocols following the scheme specified by Zhu, et al. [39]. Gene expression levels were quantified using the 2^−∆∆CT^ method [40]. Primers utilized in qRT-PCR analyses (Table S1) were developed with Premier 5.0 software (Premier Biosoft, Palo Alto, CA, Canada). These primers demonstrated PCR amplification efficiencies between 91% and 106% (Table S1).

### 2.12. Statistical Analysis

A one-way ANOVA and a two-way ANOVA were conducted in SPSS (v26.0.0.2) to analyze result differences, with significance set at *p* < 0.05. Normalized Ct values from qPCR were used to calculate transcription level fold changes via the Relative Expression Software Tool (REST, v2.0.13) [41].

## 3. Results

### 3.1. Root Morphology

**Figure 1:**
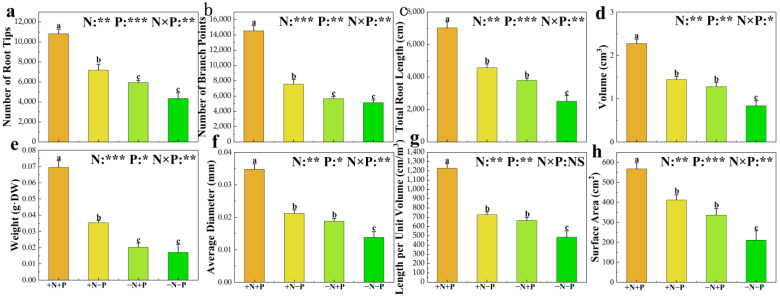
Number of Root Tips (**a**), Number of Branch Points (**b**), Total Root Length (**c**), Volume (**d**), Weight (**e**), Average Diameter (**f**), Length per Unit Volume (**g**) and Surface Area (**h**) of 84 K poplar subjected to varying nitrogen and phosphorus treatments. The data are shown as averages ± standard error (SE) with a sample size of 5. Distinct letters atop the bars denote significant differences among treatments (*p* < 0.05). *p*-values obtained from the two-way ANOVAs for nitrogen treatments (N), phosphorus treatments (P), and their interactions (N × P) are indicated. * indicates *p* < 0.05; ** indicates *p* < 0.01; *** indicates *p* < 0.001; NS, not significant.

Soil N and P availability significantly affect plant root growth and development. In our study, we analyzed the impact of varying treatments on the biomass and morphology of the poplar roots (Figure 1). NO_3_^−^ supplementation, regardless of Pi presence, significantly enhanced several parameters, including the number of root tips, branches, total root length, volume, surface area, the ratio of Length per Unit Volume, Average Diameter, and weight. In addition, Pi addition and NO_3_^−^ led to significant increases in root weights, number of tips, branches, total root length, volume, surface area, Length per Unit Volume, Average Diameter, and overall biomass. Conversely, in low NO_3_^−^ availability environments, Pi supplementation alone notably improved the total root length, volume, surface area, Length per Unit Volume, and Average Diameter.

### 3.2. N and P Content, Concentration, and Utilization Efficiency

**Figure 2:**
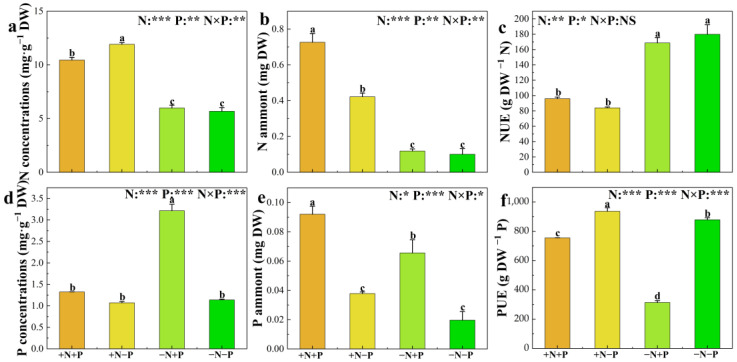
N concentrations (**a**), N amount (**b**), nitrogen utilization efficiencies (NUEs) (**c**), P concentrations (**d**), P amount (**e**), and phosphorus utilization efficiencies (PUEs) (**f**) of 84 K poplar exposed to different N and P treatments. The data are shown as averages ± standard error (SE) with a sample size of 5. Distinct letters atop the bars denote significant differences among treatments (*p* < 0.05). *p*-values obtained from the two-way ANOVAs for nitrogen treatments (N), phosphorus treatments (P), and their interactions (N × P) are indicated. * indicates *p* < 0.05; ** indicates *p* < 0.01; *** indicates *p* < 0.001; NS, not significant.

The impact of soil N and P availability on plant N and P content, concentration, and utilization efficiency is detailed in Figure 2. This study demonstrates that in low NO_3_^−^-availability environments, adding Pi significantly elevates P concentration and content in roots, notably reducing phosphorus utilization efficiencies (PUEs). Moreover, Pi addition does not affect the N concentration and content or NUE. Incorporating P into the system substantially increases P and N content when NO_3_^−^ is supplied. However, it leads to a significant decrease in N concentration and PUE without affecting the concentration of P and nitrogen utilization efficiencies (NUEs). Conversely, in low Pi availability environments, adding NO_3_^−^ markedly enhances N concentration, N content, and PUE, but reduces NUE. NO_3_^−^ alone does not significantly impact P concentration or content. With Pi available, the introduction of N significantly improves N concentration, N content, and P concentration and content, alongside PUE, yet results in a considerable reduction in NUE.

### 3.3. Enzymatic Activities in Nitrogen and Phosphorus Assimilation

**Figure 3:**
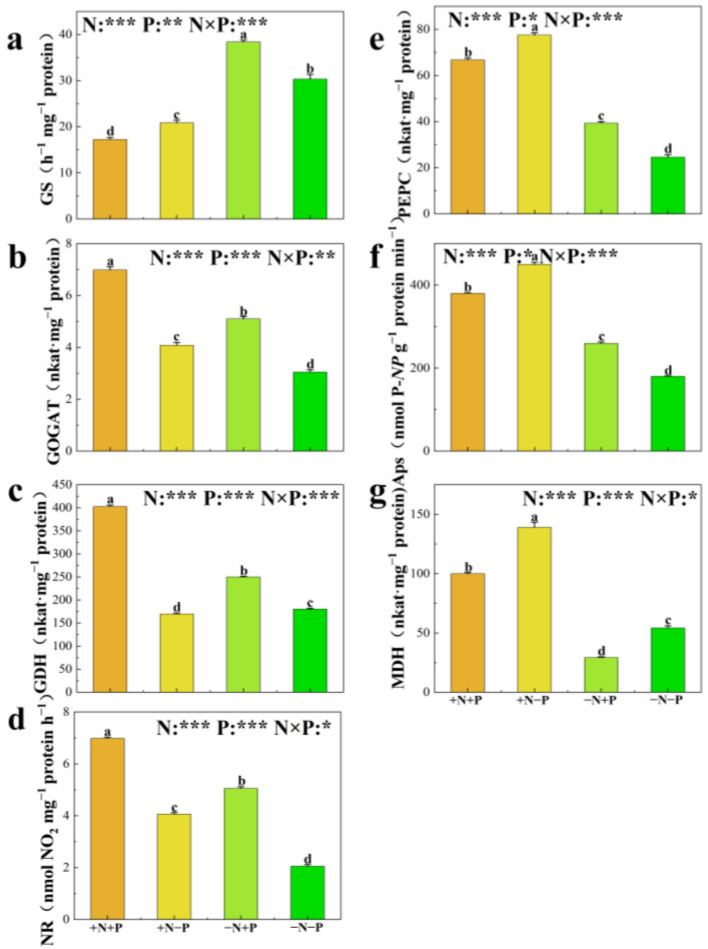
Glutamine synthetase (GS) (**a**), glutamate synthase (GOGAT) (**b**), glutamate dehydrogenase (GDH) (**c**), nitrate reductase (NR) (**d**), phosphoenolpyruvate carboxylase (PEPC) (**e**), phosphatases (Aps) (**f**), and malate dehydrogenase (MDH) (**g**) of 84 K poplar exposed to different N and P treatments. The data are shown as averages ± standard error (SE) with a sample size of 5. Distinct letters atop the bars denote significant differences among treatments (*p* < 0.05). *p*-values obtained from the two-way ANOVAs for nitrogen treatments (N), phosphorus treatments (P), and their interactions (N × P) are indicated. * indicates *p* < 0.05; ** indicates *p* < 0.01; *** indicates *p* < 0.001; NS, not significant.

The activities of phosphatases (APs), phosphoenolpyruvate carboxylase (PEPC), malate dehydrogenase (MDH), glutamine synthetase (GS), glutamate synthase (GOGAT), glutamate dehydrogenase (GDH), and nitrate reductase (NR) in poplar roots were measured (Figure 3). In low NO_3_^−^ availability environments, the addition of Pi stimulated the activities of APs, PEPC, GS, GOGAT, GDH, and NR in the roots, significantly increasing their activities by 44%, 60%, 27%, 67%, 39%, and 145%, respectively; it also significantly reduced the activity of MDH by 46%. In NO_3_^−^-supplied treatments, Pi addition notably enhanced GOGAT, GDH, and NR activities by 71%, 137%, and 62%, respectively, while slightly reducing APs, PEPC, MDH, and GS activities by approximately 16%, 144%, 28%, and 17%. The +N, −P treatment significantly enhanced root activities of APs, PEPC, MDH, GOGAT, and NR by approximately 150%, 215%, 156%, 34%, and 98%, respectively, compared to the -N,-P treatment, while slightly reducing GS and MDH activities by about 31% and 6%, respectively. The root activities of APs, PEPC, MDH, GOGAT, GDH, and NR were significantly higher in the +N, +P treatment group compared to the −N, +P group. The GS activity in roots was notably reduced in the +N, +P treatment group relative to the −N, +P treatment group.

### 3.4. Concentrations of Carbohydrate, Organic Acid

**Figure 4:**
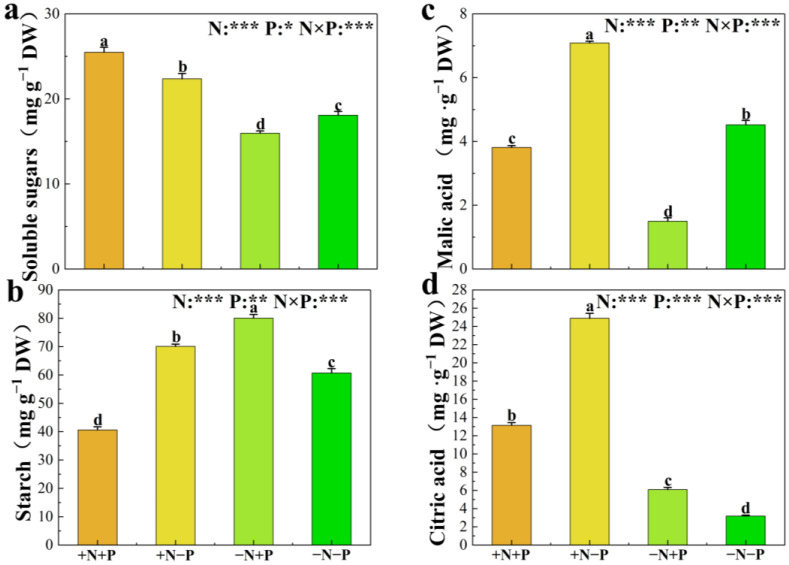
Soluble sugars (**a**), starch (**b**), malic acid (**c**), and citric acid (**d**) levels in 84 K poplar under varying N and P treatments. The data are shown as averages ± standard error (SE) with a sample size of 5. Distinct letters atop the bars denote significant differences among treatments (*p* < 0.05). *p*-values obtained from the two-way ANOVAs for nitrogen treatments (N), phosphorus treatments (P), and their interactions (N × P) are indicated. * indicates *p* < 0.05; ** indicates *p* < 0.01; *** indicates *p* < 0.001; NS, not significant.

Soluble sugar and starch are important metabolic and storage substances in plants, and the effects of different N and P treatments on their contents are significantly different (Figure 4a,b). The addition of NO_3_^−^ increased the concentration of sugar regardless of the availability of Pi, and the addition of Pi increased the concentration of sugar only when the supply of NO_3_^−^ was present. On the contrary, adding Pi significantly decreased sugar concentration in low NO_3_^−^ availability environments. The −N, +P treatment increased starch concentrations by 32%, while the +N, −P treatment resulted in a 15% increase, compared to the −N, −P treatment. The +N,+P treatment decreased starch concentration by 42% compared to the −N +P treatment and by 49% compared to the +N, −P treatment.

The nutritional status of N and P influences the synthesis and secretion of organic acids in plants. Consequently, we measured the concentrations of malic and citric acids in the roots of 84 K poplar (Figure 4c,d). The presence of NO_3_^−^ notably elevated malic and citric acid levels, with increases of 57% and 682%, respectively, in low Pi availability environments. Under Pi supply, these concentrations rose by about 155% and 116%, respectively (Figure 4c,d). Furthermore, the +N, +P treatment significantly reduced malic acid and citric acid concentrations by 46% and 47%, respectively, compared to the +N, −P treatment. The −N, +P treatment significantly reduced malic acid concentrations by 67% compared to −N, −P treatment, resulting in a notable 91% increase in citric acid concentrations.

### 3.5. Phytohormone Concentrations

**Figure 5:**
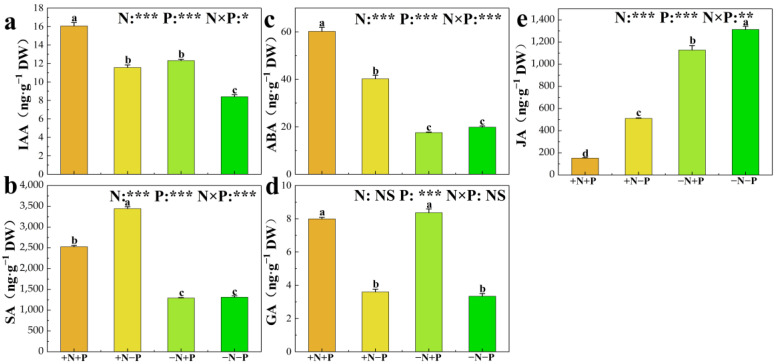
Indole-3-acetic acid (IAA) (**a**), salicylic acid (SA) (**b**), abscisic acid (ABA) (**c**), gibberellin (GA_3_) (**d**), and jasmonic acid (JA) (**e**) of 84 K poplar exposed to different N and P treatments. The data are shown as averages ± standard error (SE) with a sample size of 5. Distinct letters atop the bars denote significant differences among treatments (*p* < 0.05). *p*-values obtained from the two-way ANOVAs for nitrogen treatments (N), phosphorus treatments (P), and their interactions (N × P) are indicated. * indicates *p* < 0.05; ** indicates *p* < 0.01; *** indicates *p* < 0.001; NS, not significant.

Indole-3-acetic acid (IAA), salicylic acid (SA), jasmonic acid (JA), abscisic acid (ABA), and gibberellin (GA_3_) are crucial for root system growth and development. This study quantifies the concentrations of these hormones in roots, as illustrated in Figure 5. In low Pi availability environments, the addition of NO_3_^−^ significantly increased the concentrations of IAA, SA, and ABA by 38%, 162%, and 103%, respectively, and significantly decreased the concentrations of JA by 61% (Figs. 5). The addition of NO_3_^−^ also increased significantly concentrations of IAA, SA and ABA by 30%, 95%, and 243%, respectively, and significantly decreased the concentrations of JA by 86% when Pi was available. Meanwhile, these treatments had no significant effect on GA_3_ concentration. In low NO_3_^−^-availability environments, adding Pi significantly increased the concentrations of IAA and GA_3_ by 47% and 151%, respectively, and significantly decreased the concentrations of JA by 14%. However, the above treatment did not obviously affect SA and ABA concentrations. Adding Pi increased considerably the concentrations of IAA, ABA, and GA_3_ by 39%, 50%, and 122%, respectively, and significantly decreased the concentrations of SA and JA by 27% and 70% when Pi was available.

### 3.6. Transcriptome Sequencing Datasets

We performed genome-wide RNA sequencing to investigate the transcriptomic regulation of poplar root morphology and physiology in response to N and P interactions, generating 112 million raw reads across libraries, each yielding from 42.8 to 51.8 million reads. Sequence trimming resulted in from 42.3 to 51.4 million clean reads per library. Between 38.5 and 49.9 million clean reads per library were mapped to the *P. alba* genome, with mapping ratios ranging from 88.42% to 97.43% (Table S2).

In comparison to the roots without N or P (−N, −P), roots exposed to NO_3_^−^ application (+N, −P vs. −N, −P) showed a significant increase in the transcript levels of 891 genes, while 347 genes exhibited a significant decrease (Figure S1). In response to Pi application, the expression levels of 615 genes increased, while those of 559 genes decreased (−N, +P vs. −N, −P, Figure S1). Upon NO_3_^−^ application, compared to the roots with Pi only (−N, +P), transcript levels of 1298 genes increased, whereas 1791 genes showed a decrease (+N, +P vs. -N, +P, Figure S1). Finally, when compared to roots with only NO_3_^−^ application (+N, −P), 333 genes displayed increased transcript levels in response to Pi application, while 163 genes decreased (+N, +P vs. +N, −P, Figure S1).

**Figure 6:**
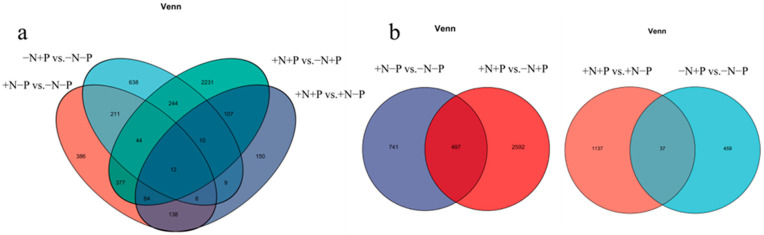
DEGs in the roots of 84 K poplar under varying N and P treatments. (**a**,**b**) The shared and distinct genes between the contrasting groups in the roots. A Venn diagram was created using the Calculate and Construct custom Venn diagrams tool.

In addition, 1633 genes showed a regulatory response to Pi application under different NO_3_^−^ conditions, 1137 genes showed a regulatory response to Pi supply only under NO_3_^−^ supply, and 459 genes showed a regulatory response to Pi supply under no NO_3_^−^ supply conditions. In addition, 37 genes exhibited regulatory responses to both treatments (Figure 6a,b).

Among them, 3830 genes showed a regulatory response to NO_3_^−^ application under different Pi conditions, 2592 genes showed a regulatory response to NO_3_^−^ supply only under Pi supply, and 497 genes showed a regulatory response to NO_3_^−^ supply under no Pi application conditions (Figure 6a,b).

### 3.7. Functional Enrichment Analysis of Differentially Expressed Genes (DEGs)

We utilized Blast2GO to identify significant Gene Ontology (GO) terms, thereby improving our understanding of the functions linked to upregulated and downregulated genes in poplar roots across the following comparisons: (+N,−P vs. −N,−P), (−N,+P vs. −N,−P), (+N,+P vs. −N,+P), and (+N,+P vs. +N,−P). To clarify the differential gene expression profile under varying nutrient conditions in poplar roots, we investigated the enriched GO terms of DEGs in response to NO_3_^−^ supplementation with and without Pi deficiency. The results indicated that upregulated DEGs under NO_3_^−^ application, with concurrent Pi application, were prominently enriched in processes such as “nitrate metabolic process”, “phospholipid catabolic process”, and “negative regulation of cytokinin-activated signaling pathway”. (Figure S2, Table S2). Conversely, downregulated DEGs were primarily enriched in the subcategories “phosphatidylcholine biosynthetic process”, “adenylylsulfate kinase activity”, and “auxin-activated signaling pathway”. In the “+N,+P vs. −N,+P” comparison, most upregulated DEGs were enriched in “xyloglucan metabolic process”, “auxin homeostasis”, and “cell wall polysaccharide metabolic process”, while downregulated DEGs were enriched in “jasmonic acid biosynthetic process”, “organophosphate catabolic process”, and “phospholipid dephosphorylation” (Figure S2, Table S2). Significant differences were also identified regarding DEG-enriched GO terms in response to Pi supplementation with or without NO_3_^−^ deficiency. In the “−N,+P vs. −N,−P” comparison, upregulated DEGs were largely enriched in “phosphorus metabolic process”, “phosphate-containing compound metabolic process”, and “small molecule metabolic process”, while downregulated DEGs were enriched in “biosynthetic process”, “polysaccharide metabolic process”, and “flavonoid metabolic process” (referenced in Figure S2, Table S2). In the “+N,+P vs. +N,−P” comparison, upregulated DEGs were enriched in “glutamate dehydrogenase (NAD+) activity”, “peptidase inhibitor activity”, and “amine biosynthetic process”, while downregulated DEGs were primarily enriched in “cellular response to phosphate starvation”, “ferric iron binding”, and “phosphatase activity” (Figure S2, Table S2).

A KEGG (Kyoto Encyclopedia of Genes and Genomes) pathway analysis was conducted to systematically evaluate the metabolic pathways associated with DEGs. The analysis indicated that roots, when exposed to either Pi or NO_3_^−^, respectively, were significantly enriched in 17 pathways compared with the −N,−P control group. Additionally, in the +N,+P treatment group, DEGs were significantly enriched in 23 or 14 pathways, respectively, compared to the +P,−N or −N,+P treatment groups (Figure S3). Furthermore, in the +N,−P vs. −N,−P comparison, most DEGs were predominantly enriched in pathways such as “Tropane, piperidine, and pyridine alkaloid biosynthesis”, “Amino sugar and nucleotide sugar metabolism”, and “Inositol phosphate metabolism” (Figure S3). In the +N,+P vs. −N,+P comparison, enriched pathways included “Biosynthesis of various secondary plant metabolites”, “Plant hormone signal transduction”, and “Starch and sucrose metabolism” (Figure S3). For the −N,+P vs. −N,−P comparison, significant enrichment was observed in “Flavonoid biosynthesis”, “Tropane, piperidine, and pyridine alkaloid biosynthesis”, and “Starch and sucrose metabolism” (Figure S3). Lastly, in the +N,+P vs. +N,−P comparison, the main enriched pathways were “Glycerophospholipid metabolism”, “Plant hormone signal transduction”, and “Inositol phosphate metabolism” (Figure S3).

### 3.8. Functional Categorization of Transcriptional Factors (TFs) and Comparative Analysis

Recognizing the importance of TFs in gene expression regulation and plant physiological responses to environmental stress, we thoroughly analyzed the TFs among the DEGs.

Our detailed analysis of the data revealed that the DEGs contained various TF families, including AP2, B3, bHLH, bZIP, C3H, CO-like, Dof, EIL, ERF, GATA, GRAS, GRF, HB-other, M_type, MYB, MYB_related, NAC, NF-YA, Nin-like, TCP, WRKY, ZF-HD, ARF, DBB, HSF, LBD (AS2/LOB), TALE, C2H2, BES1, CPP, HD-ZIP, LSD, MIKC, NF-YB, SBP, and Trihelix. In addition, specific, unspecified, and putative DNA-binding domain TFs were identified. The data showed that 310 DEGs involving 32 TF families or 28 DEGs involving 9 TF families were putatively related to TFs, respectively, in the +N,+P treatment group compared to the −N,+P or +N,−P treatment group (Figure S4b,d). Compared with those in the −N,−P treatment group, 99 involving 17 TF families or 91 DEGs involving 22 TF families were putatively related to TFs, respectively, under the +N,−P or −N,+P conditions (Figure S4a,c).

### 3.9. Validation of RNA-Seq Analysis

To validate the sequencing results, we selected 12 DEGs for further analysis by qRT-PCR. The calculated Pearson’s correlation coefficient (R^2^ = 0.88) indicated a high concordance between the two techniques and, therefore, a high reliability of the data presented in this study (Figure S5).

## 4. Discussion

### 4.1. The Supply of Nitrates and Phosphates Regulates the Plasticity of the Root System Through Hormonal Networks

Plant hormones, including IAA, ABA, GA, JA, and SA, play a key role in regulating the morphology of the root system in adapting to N and P nutritional conditions. Studies have shown that these hormones regulate root length, surface area, and the number of branches in response to changes in various nutritional levels [42,43,44,45,46,47,48]. The accumulation of auxin fosters root development, thereby augmenting the plant’s capacity for nutrient uptake [49]. Specifically, nitrate regulates plant auxin concentrations by modulating the expression of genes involved in auxin biosynthesis (e.g., *TAA1*, *TARs*, *YUCs*), auxin conjugation (*GH3* family), and auxin transport (*AUXs*, *LAXs*, *PINs*) [50,51,52]. Our research indicates that, in low Pi availability environments, the supply of NO_3_^−^ leads to a decrease in the transcription levels of *ARF*, *AUX/IAA*, *PILS3*, and *ILL6*, while increasing the transcription of the IAA biosynthesis gene *YUC6*. This may be the primary reason for the increased IAA concentration in the roots. It has been documented that MYB52 negatively impacts the transcription of critical genes within the IAA biosynthetic pathway [53]. Our results indicate that, in conditions of Pi availability, NO_3_^−^ supply may enhance IAA synthesis by the downregulation of *MYB52*, consequently increasing IAA concentrations. Furthermore, N and P nutrition significantly influence JA metabolism. Our findings show that, in low Pi availability environments, NO_3_^−^ supply substantially reduces the transcription levels of *JOX2* and *MYC2*, while simultaneously enhancing the transcription of *MJE1*. Upon Pi supplementation, a decrease in the mRNA levels of *JOX2*, *JAZ*, and *MYC2*, along with an elevation in *MJE1* transcription, potentially results in lower root JA concentrations. N is a key regulator of ABA biosynthesis and transport during root development, with ABA biosynthesis positively correlating with elevated root NO_3_^−^ levels. Notably, nitrate facilitates ABA accumulation in the endodermis and quiescent center to sustain meristem activity and upregulates *β-glucosidase 1* (*BG1*) expression, which releases active ABA from ABA-glucosyl ester (ABA-GE) [13]. Our results imply that NO_3_^−^ supplementation boosts *BG1* transcription, potentially leading to elevated concentrations of ABA in the roots. Recent research underscores the interaction between nitrate and other plant hormones, including GA and SA. It has been shown that DELLA proteins modulate MYC2’s functions via interactions, thereby exerting complex regulatory influence within the jasmonic acid signal pathway [54]. Our research reveals that NO_3_^−^ supplementation enhances the transcription of *DELLA* in the GA pathway, potentially providing negative feedback for the increased expression of *MYC2*, thereby affecting root cell differentiation. In conclusion, NO_3_^−^ supplementation significantly increases root IAA, SA, and ABA concentrations while mitigating JA levels by modulating the expression of genes within various hormone pathways. Rising IAA and ABA levels, coupled with elevated expression of key pathway genes, potentially facilitate root elongation, surface area expansion, and branching, enhancing nutrient acquisition. Additionally, elevated ABA levels benefit drought resistance, and increased SA levels may enhance N absorption via nitrate metabolism enhancement. Decreased JA concentrations might lower defense mechanism activation, allocating more resources to growth and possibly contributing to enhanced root biomass.

**Figure 7:**
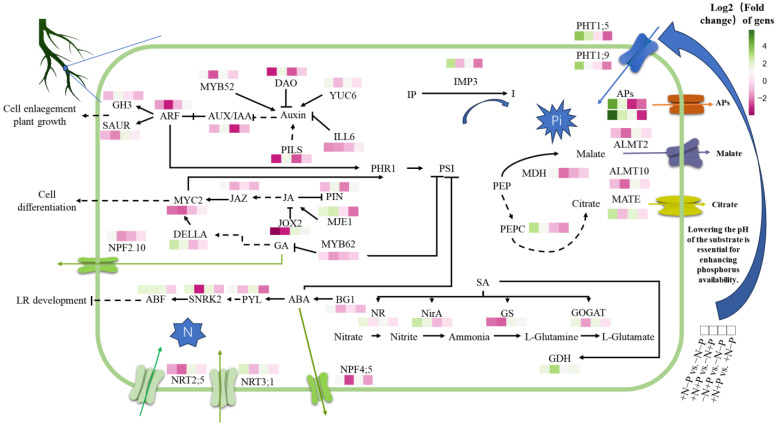
Comprehensive model of nitrogen and phosphorus interaction at different levels. +N,−P vs. −N,−P (first box on the left), +N,+P vs. −N,+P (second box on the left), −N,+P vs. −N, −P (middle box), and +N,+P vs. +N,−P (first box on the right). In the metabolic pathway, a solid arrow indicates a direct step, while a dotted arrow signifies an indirect step. On the color scale, green indicates an increase, while red indicates a decrease. Inositol Phosphates (IPs), Inositol (I), Phosphoenolpyruvate (PEP), Multidrug and Toxic Compound Extrusion (MATE), Aluminum-activated Malate Transporter (ALMT), Acid Phosphatases (APs), Nitrite Reductase A (NirA).

Adequate Pi supply enhances concentrations of plant hormones like IAA and GA, promoting plant growth [55]. Our findings indicate that, without NO_3_^−^, Pi supplementation may promote the production of IAA by decreasing the transcription levels of *PIN2*, *PILS3*, and *AUX/IAA*, while simultaneously increasing the transcription levels of *SAUR* and *YUC6*. The upregulation of *SAUR* will benefit the development of roots and hypocotyls, improving nutrient absorption [56,57]. Notably, the impact of Pi on auxin metabolism hinges on the availability of NO_3_^−^, as Pi supply without NO_3_^−^ does not significantly alter gene expression related to auxin metabolism (Figure 7). Additionally, Pi supplementation under low NO_3_^−^ availability environments augments the transcription levels of *PYL*, *SNRK2*, and *ABF*, potentially inhibiting lateral root development [58]. However, with adequate NO_3_^−^, Pi’s influence on ABA signaling pathway genes is minimal, though it does promote ABA accumulation in roots. In the JA signaling pathway, increased JA concentration indirectly stimulates *MYC2* transcription [59]. Our results show that Pi supply significantly enhances *MYC2* transcription levels only in the presence of NO_3_^−^, indicating that Pi’s impact on *MYC2* transcription might depend on NO_3_^−^ availability and suggesting JA accumulation in roots. Research indicates that overexpression of *MYB62* in *Arabidopsis* suppresses early gibberellin biosynthesis genes, leading to GA deficiency [60]. Our data demonstrate that Pi addition notably raises *MYB62* transcription, implying that the observed GA concentration increase with Pi supplementation is partly due to *MYB62* upregulation. Furthermore, since SA bolsters nitrogen metabolism by enhancing nitrogenase activity [61], an increase in SA from Pi supplementation in the presence of sufficient NO_3_^−^ could boost nitrogen metabolism by augmenting nitrogenase activity (Figure 7). In conclusion, in low NO_3_^−^ availability environments, Pi supply markedly improves the accumulation of IAA and GA_3_ in poplar roots, thus enhancing root system length, volume, surface area, and specific root length, and increasing average root diameter, thereby improving nitrogen and P absorption. Additionally, the reduced JA concentration might mitigate the defense metabolic burden induced by N deficiency to some extent. With available NO_3_^−^, Pi supply further increases ABA concentration, promoting the development of a complex and refined root architecture, increasing root branching density and specific surface area, thus facilitating the absorption of N and P nutrients from soil.

In conclusion, NO_3_^−^ and Pi can independently enhance poplar root architecture by promoting the accumulation of IAA and ABA, as well as IAA and GA_3_, thus broadening the physical absorption surface and enhancing nutrient absorption. However, the simultaneous decrease in JA concentration resulting from both inputs might partially impair the plant’s defense mechanisms against biotic stresses and its adaptability to abiotic stresses.

### 4.2. Regulation of Plant Metabolic Networks Under Nitrogen and Phosphorus Interaction

Plants generate NH_4_^+^ from NO_3_^−^ via NR and nitrite reductase [62]. Subsequently, GS converts NH_4_^+^ into glutamine, while GOGAT and GDH transform it into glutamate. The activity of NR, GOGAT, and GDH enzymes in poplar roots and leaves declines with low nitrogen levels but rises as nitrogen availability increases [25,63]. Data indicate that, in low NO_3_^−^ availability environments, Pi supply markedly increases the transcription levels and activities of GDH, GOGAT, and GS, along with NR, which may trigger N assimilation potential via phosphorization signaling or energy sources like starch accumulation. However, N deficiency, resulting in scarce nitrate substrates, renders the enzyme system ineffective, failing to assimilate nitrogen and leading to unchanged nitrogen content. This scenario of high enzymatic activity but low efficiency highlights the imbalance in P-driven N metabolism compensation. While Pi facilitates the expression of N assimilation enzymes through carbon metabolism, the lack of a NO_3_^−^ source disrupts the metabolic pathway, leading to a noticeable decline in PUE and waste of N metabolism resources. Hence, while P fertilizer alone can momentarily boost N metabolism potential in N-deficient soil, pairing it with N supplementation is critical for the enzyme system’s functional transformation, thus avoiding the metabolic dilemma of “high P, low N”.

PEPC, APs, and MDH are pivotal in both acquiring and assimilating phosphorus [64,65,66]. APs, functioning both extracellularly and intracellularly, are crucial for the uptake of exogenous Pi from the soil and for catalyzing the hydrolysis of various phosphomonoesters [67]. In conditions of Pi scarcity, AP expression markedly increases, thereby boosting the activity of AP enzymes outside and inside the cell [64]. PEPC facilitates the conversion of phosphoenolpyruvate (PEP) into oxaloacetate (OAA), with MDH subsequently catalyzing its transformation into malate. This malate, derived from citrate via glycolysis and the tricarboxylic acid cycle (TCA), is excreted into the rhizosphere soil, where it chelates metal ions like Ca^2+^ and Al^3+^. This action aids Pi fixation and enhances its soil availability, a process critical for plants to adapt to phosphorus scarcity [68,69]. Therefore, increasing soil P concentration is essential for plant adaptation to P deficiency.

Our findings reveal that NO_3_^−^ supplementation significantly enhances the transcription levels of MDH, PEPC, and APs, notably in low Pi availability environments, which likely increases the production of these enzymes. Additionally, NO_3_^−^ supply boosts the enzymatic activities of MDH, PEPC, and APs, potentially improving P metabolism. NO_3_^−^ addition raises malate and citrate levels in roots, leading to heightened carbon utilization and a substantial decrease in soluble sugar levels. Even though the precise concentrations of malate and citrate in root exudates remain unquantified, the elevated mRNA expression of citrate transporter genes (*MATEs*) under low Pi availability environments implies that NO_3_^−^ supply may augment citrate secretion. However, in Pi-adequate conditions, NO_3_^−^ supply does not significantly impact the mRNA levels of *MATE* but markedly reduces the transcription of malate transporters (*ALMTs*) (Figure 7). This reduction likely occurs because ample P negates the need for pH modification of the substrate through excessive acid secretion. Conversely, the absence of NO_3_^−^ attenuates the negative regulatory effect of P on transporter proteins. Likewise, in low Pi availability environments, the addition of NO_3_^−^ does not substantially influence *ALMT* gene transcription. However, Pi availability induces a notable increase in malate transporter gene activity, potentially augmenting malate efflux. Based on the optimal resource allocation theory, plants assign biomass to the most resource-constrained organs [70,71,72]. Although higher organic acid levels increase carbon expenditure, our research suggests that NO_3_^−^ supplementation enriches soluble sugar accumulation in roots while diminishing starch reserves, highlighting a primary resource allocation mechanism. In contrast to poplar trees, which rely on PEPC/MDH for malate secretion, subtropical trees predominantly enhance phosphorus acquisition under low phosphorus conditions by elevating root phosphatase activity [73]. Meanwhile, Proteaceae adopt a combined strategy to activate soil phosphorus when facing phosphorus scarcity [74]. Despite the variance in metabolite types, woody plants universally facilitate P activation via optimized carbon allocation, such as reducing starch accumulation. This suggests that a ‘carbon-P synergy’ represents a unified strategy across different species.

In conclusion, NO_3_^−^ supplementation may enhance the production and secretion of organic acids by upregulating the transcription and enzymatic activities of MDH and PEPC, as well as increasing both the concentration of organic acids in roots and the transcription levels of their transporter proteins. This process is complemented by the concurrent elevation of APs’ transcription and enzymatic activities, contributing to a rise in the available Pi concentration in the substrate. Ecologically, judicious use of nitrogen fertilizer may facilitate P uptake, whereas excessive NO_3_^−^ application risks soil acidification, P fixation, and increased eutrophication in aquatic systems. Nevertheless, precise adjustment of the N-P balance, coupled with leveraging the plant’s enzyme mechanics to boost P activation, can mitigate reliance on P fertilizers.

### 4.3. Synergistic Regulation of Molecular Mechanisms in Nutrient Absorption by N and P Interaction in Plants

Longer fine roots and expanded root surface areas enhance the uptake of Pi by plants, with the augmentation of primary Pi transport mechanisms playing a crucial role in boosting phosphorus absorption efficiency [75,76,77]. Our research demonstrates that NO_3_^−^ supplementation significantly enhances the expression levels of Pi transporters, particularly when Pi is deficient, facilitating a greater expression of these transporters (Figure 7). Although low phosphorus levels naturally stimulate Pi transporter expression [78], this regulatory effect by NO_3_^−^ diminishes with increased Pi supply, leading to the down-regulation of certain phosphate transporters (Figure 7). Intriguingly, the inhibitory effect of Pi on Pi transporter expression is contingent on the presence of NO_3_^−^. In the absence of NO_3_^−^, Pi induces the expression of only a few Pi transporters and suppresses the expression of *PHO1*;*H3*.

In conditions of P scarcity, plants hydrolyze phosphorus-rich organic compounds, including inositol phosphates, phospholipids, and phosphoenolpyruvate (PEP), to recover phosphates. Lipid remodeling and the decomposition of organophosphates are metabolic reactions to phosphorus starvation [79,80]. Our data indicated that, in low Pi availability environments, increasing NO_3_^−^ supply elevated the transcription level of *IMP3*, enhanced phosphoinositide degradation, and facilitated P recovery. Conversely, when N was abundant, Pi supplementation down-regulated *IMP3* transcription and exerted a negative regulatory effect (Figure 7). Studies have demonstrated that inositol pyrophosphates (PP-InsPs), acting as nutrient messengers, critically influence phosphorus signaling through inhibiting PHOSPHATE STARVATION RESPONSE2 (PHR2)—by activating Phosphate Starvation-Induced (PSI) gene expression by mediating the SPX-PHR2 complex formation. Under low Pi conditions, PP-InsPs decompose, leading to the dissociation of SPX and PHR2, thereby allowing PHR2 to enter the nucleus and activate PSI gene expression [81,82,83]. Additionally, NLA1 has been shown to mediate PHR1 ubiquitination in a PP-InsPs-dependent manner [84]. The biosynthesis of PP-InsPs in plants is primarily catalyzed by inositol tetrakisphosphate kinase (ITPK) and diphosphoinositol pentakisphosphate kinase (VIH). In *Arabidopsis*, ITPK1 phosphorylates InsP_6_ to produce InsP_7_, a process dependent on the phosphate concentration at the 5-position, subsequently leading to the degradation of 5-InsP_7_ [85]. ITPK1 phosphorylates InsP_6_ to generate InsP_7_ and mediates the decomposition of 5-InsP_7_, a process that Pi concentration affects [86]. Our findings indicated that providing NO_3_^−^ reduced the transcription of *ITPK1* and the production of PP-IPs when Pi was supplemented. Supplying NO_3_^−^ under low Pi availability environments may boost PSI gene expression and augment Pi absorption via the facilitation of PP-IP_S_ degradation, achieved through upregulating *ITPK1* transcription. Consequently, PP-InsPs might be crucial in mediating the interaction between N and P signaling in poplar.

Elevated IAA levels facilitate cell wall-associated phosphorus recycling, mitigating Pi scarcity [87]. As noted, the rise in IAA concentrations induced by nitrate encourages Pi recycling. Furthermore, SA enhances soluble P levels in roots and stems through the modulation of cell wall P cycling [17,87]. Our research indicates that, in low Pi availability environments, the supply of NO_3_^−^ may improve P absorption and recycling efficiency. This improvement is achieved through the co-regulation of PHO family gene expression, activation of the inositol phosphate metabolism pathway (notably by upregulating *IMP3* and *ITPK1* to aid PP-InsPs degradation), and the mediation of P cycling via hormone signals controlled by IAA and SA. Moreover, it might maintain N-P metabolic balance under conditions of elevated P through a feedback inhibition mechanism.

NRT2;5 is activated under prolonged nitrogen starvation conditions, forming an integral part of a high-affinity transport system [88]. Together, NRT2;1 and NRT3;1 constitute a high-affinity nitrate transport complex [89] that is crucial for plant nitrate absorption. Studies have demonstrated that phosphorus deficiency impedes nitrogen assimilation and absorption in plants [90], leading to nitrogen accumulation in plant roots, thereby inhibiting nitrogen absorption via a feedback mechanism [91]. Our findings corroborate this observation. Specifically, Pi availability modulates the impact of NO_3_^−^ on the expression of NO_3_^−^ transport proteins; under normative conditions, only NO_3_^−^ supply reduces the mRNA levels of *NRT2;5*. However, in conditions lacking phosphorus, NO_3_^−^ availability elevates the mRNA levels of *NRT2;1* and *NRT3;1*. While NO_3_^−^-rich environments minimally affect the mRNA levels of NO_3_^−^ transport proteins with added Pi, Pi supplementation in NO_3_^−^-deficient scenarios boosts the mRNA levels of *NRT2;1*, *NRT2;5*, and *NRT3;1*, thus enhancing NO_3_^−^ absorption in plants (Figure 7). Additionally, NO_3_^−^ transport proteins NPF3 and NPF2.10 have been identified as gibberellin transporters, contributing to the N and GA interplay [92,93]. Our analysis reveals that only in the presence of Pi, the transcriptional response of *NPF2.10* to NO_3_^−^ diminishes, indicating Pi’s potential to impair NO_3_^−^ transport by hindering *NPF2.10* transcription. Pi regulation disrupts NO_3_^−^ absorption in its absence, yet its supply could bolster plant nitrate assimilation by upregulating these transport proteins (Figure 7). In poplar trees, Pi availability may affect N uptake through the regulation of gene expression in the high-affinity NRT2.1/NRT3.1 system and the NPF family. Pi activates the NO_3_^−^ transport system in low N availability environments, whereas it triggers negative feedback by inhibiting the expression of transport proteins such as NPF2.10 when N is plentiful.

In summary, in low Pi availability environments, NO_3_^−^ significantly enhances the plant’s ability to absorb and recycle P. This enhancement occurs through a synergistic upregulation of P transport proteins (e.g., the PHO family), activation of inositol phosphate metabolism (through IMP3/ITPK1-mediated degradation of PP-InsPs), and stimulation of hormone signaling pathways (IAA/SA promoting P cycling). Concurrently, Pi intricately modulates the activity of NO_3_^−^ transport proteins—activating the high-affinity NRT2.1/NRT3.1 system in low N scenarios and inhibiting NPF2.10 in high N environments. This dynamic regulation might be pivotal in the N absorption’s negative feedback mechanism and in sustaining the metabolic equilibrium between N and P.

### 4.4. The Interaction Between N and P Regulates a Cascading Network of TFs

Both NO_3_^−^ and Pi affect the expression level of transcription factors. In this study, we analyzed and identified many TFs (Figure S4). As shown in Figure S6, the expression of most transcription factors is regulated by NO_3_^−^ and Pi.

**Figure 8:**
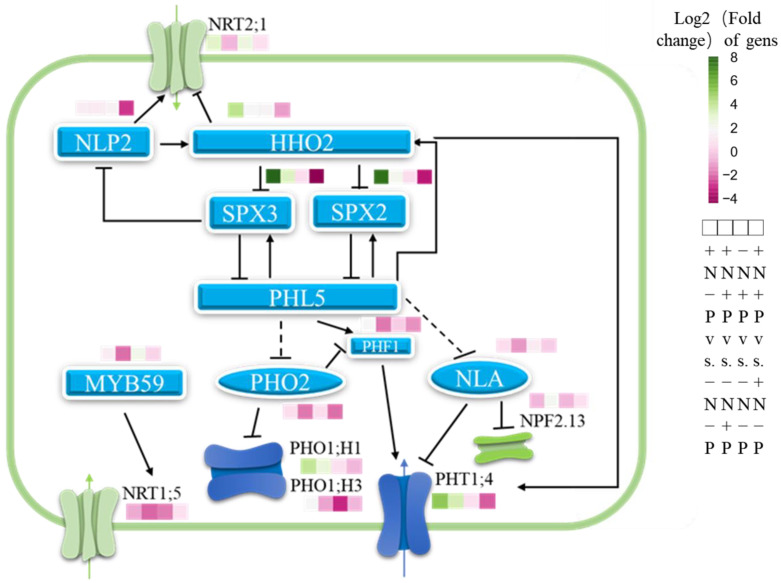
The schematic diagram model of interactive regulation of nitrogen and phosphorus in 84 K poplar. +N,−P vs.-N,−P (first box on the left), +N,+P vs. −N,+P (second box on the left), −N,+P vs. −N,−P (middle box), and +N,+P vs. +N,−P (first box from the right). Nitrogen and phosphorus regulate each other’s absorption and metabolism through several core crosstalk factors.

Research has demonstrated that SPX1 and SPX2 proteins modulate the activity of PHR2 by binding through their SPX domains, thus inhibiting its association with PIBS [94]. Moreover, analysis of genetic interactions has revealed that SPX3/5 acts as functional repressors of OsPHR2, the rice analogue of AtPHR1, which is central to Pi homeostasis and signaling [95]. PHR1, a transcription factor involved in phosphate starvation signaling, binds to PIBS to enhance the expression of Pi starvation response genes [96]. Our findings reveal that in low Pi availability environments, NO_3_^−^ supply may stimulate PSI gene expression through a significant increase in *PHR1-Like5* (*PHL5*) transcription, thereby adapting to low Pi availability environments (Figure 8). This may contribute significantly to the marked improvement in plant phosphorus utilization efficiency and the upregulation of PHO phosphate transporter family genes. Notably, *PHL5* transcription is also induced by NO_3_^−^ in the presence of Pi, potentially in response to elevated *SPX2* and *SPX3* transcription, maintaining Pi homeostasis. Additionally, when NO_3_^−^ is abundant, Pi supply downregulates the transcription of *SPX2* and *SPX3*. This will facilitate the function of PHL5, which could be a critical reason for the increased P concentration and amount in roots.

Research has demonstrated that ARFs within the IAA signaling pathway facilitate the upregulation of PHR1 [97]. The induction of ARFs by NO_3_^−^ may enhance their functionality. Additionally, the interaction between MYC2 and PHR1, which promotes JA-induced anthocyanin biosynthesis and restricts root expansion [98], might be impacted by the reduction of NO_3_^−^-triggered *MYC2* expression. This alteration could potentially increase the availability of PHR1 for activating PSR. It has been previously established that ABA exerts an inhibitory effect on several genes induced by phosphate starvation [99,100]. Therefore, the elevation of ABA concentrations, induced by NO_3_^−^, may impede the expression of these genes, negatively influencing PSR. Furthermore, the overexpression of *MYB62* in *Arabidopsis* dampens the expression of a range of PSI genes, thereby impairing phosphate absorption [60]. Our findings indicate that, in the presence of adequate P, NO_3_^−^ selectively diminishes the transcription of *MYB62*. This reduction is predictive of an enhanced expression of PSI genes, subsequently improving phosphate uptake.

PHO2 (UBC24), an E2 hydrolase, responds to Pi availability by mediating the degradation of PHO1 through vacuolar protein hydrolysis and also negatively regulates the abundance of PHF1 and PHT1 under sufficient Pi conditions by facilitating PHT1 protein degradation [101]. NLA, an E3 ubiquitin-protein ligase, governs phosphate homeostasis by directing the ubiquitination and subsequent degradation of plasma membrane-localized phosphate transporters PHT1;1 and PHT1;4 [102]. It also aids in adapting to nitrogen deficiency by regulating nitrate remobilization through the polyubiquitination and degradation of the plasma membrane-localized nitrate transporter NPF2.13/NRT1.7 [103]. Our data show that, in the presence of Pi, NO_3_^−^ downregulates the transcription of *NLA*, diminishing its post-translational inhibition of PHT1;4 and also reducing *PHO2* transcription, thereby weakening its repression of PHO1, PHF1, and PHT1;4, allowing for increased participation in Pi uptake (Figure 8). In the absence of NO_3_^−^, Pi may upregulate NLA transcription to enhance the ubiquitination and degradation of the nitrate transporter *NPF2.13*/*NRT1.7*, thereby inhibiting the absorption and transport of NO_3_^−^. In the presence of sufficient NO_3_^−^, Pi supply does not significantly affect *NLA* transcription. Low NO_3_^−^ levels induce *NLA*, so its expression is suppressed with ample NO_3_^−^ (Figure 8). NLP2 emerges as a crucial nitrate sensor and regulator in *Arabidopsis*, alongside NLP7 [104]. Our findings indicate that NO_3_^−^ and Pi supplies positively regulate *NLP2* expression levels. Nevertheless, in conditions of phosphorus or nitrogen deficiency, Pi supply exerts a negative regulatory influence. Given that SPX4 can inhibit NLP3 from entering the nucleus by forming a complex with it [105], we hypothesize that the interplay between nitrogen and phosphorus signaling pathways might impact *NLP2*’s expression and its post-transcriptional regulation.

Additionally, MYB59, a member of the MYB family of transcription factors, plays a direct role in modulating the expression of the nitrate transporter *NRT1.5/NPF7.3* by acting on its promoter region [106]. Increasing *MYB59* transcription levels aids the up-regulation of *NRT1.5* transcription and protein accumulation. Our data reveal that a NO_3_^−^ supply in low Pi availability environments significantly enhances *MYB59* transcription levels, and, conversely, Pi availability can similarly elevate these levels in the absence of NO_3_^−^. Interestingly, elevated *MYB59* expression resulted in divergent outcomes: despite Pi-induced expression augmentation, N uptake did not significantly improve due to limited NO_3_^−^ availability. This variation in *MYB59* transcription levels implies that Pi levels could potentially increase N absorption in poplar through the upregulation of MYB59. However, this regulatory effect is markedly dependent on environmental NO_3_^−^ availability.

HHO2, a transcription factor responsive to Pi deprivation, has been shown, in overexpression lines, to enhance lateral root development and Pi uptake rate; it also targets the transcription factor PHR1 [107]. Known alternatively as NIGT1.2, HHO2 upregulates the Pi transporter genes *PHT1;1* and *PHT1;4* while downregulating nitrate transporters *NRT1;1, NRT2;1*, *NRT2;4*, and *NRT2;5*, as well as *SPX* expression by binding to cis-elements in their promoters [108,109,110,111]. Additionally, NLP triggers NIGT1.2 expression, inhibiting the expression of NLP-activated nitrate metabolism genes to maintain nitrate homeostasis in plants [19]. PHRs enhance NIGT1 expression, thereby limiting nitrate uptake and competing with PHRs for the binding site of the PHR regulatory gene. Conversely, NIGT1 inhibits PSR gene expression and modulates the phosphorus uptake balance [112]. Our findings suggest that an increased supply of NO_3_^−^ notably elevates *HHO2* transcription levels, potentially facilitating improved phosphorus uptake and nitrogen equilibrium. Notably, in low Pi availability environments, an NO_3_^−^-induced elevation in *HHO2* transcription could enhance Pi absorption and suppress PSR activity, promoting P homeostasis. Conversely, Pi supplementation decreases *HHO2* transcription, potentially limiting Pi uptake while possibly increasing nitrate absorption, thus contributing to nutritional equilibrium (Figure 8). In summary, NO_3_^−^ provision upregulates *HHO2*, which is implicated in Pi uptake. Such increased expression likely bolsters Pi absorption in low Pi environments. Furthermore, nitrate may modulate the PSR by orchestrating PHR2 activity via SPX protein regulation. This regulatory mechanism plays a crucial role in reestablishing P equilibrium following depletion. Concurrently, NO_3_^−^ may facilitate increased Pi uptake by downregulating the *NLA* transcription factor and diminishing the post-translational repression of PHT1;4. In the absence of NO_3_^−^, higher Pi availability markedly upregulates *MYB59* transcription, thereby enhancing the induction of *NRT1.5*/*NPF7.3* and potentially boosting NO_3_^−^ absorption. Moreover, Pi provision downregulates *HHO2* transcription, which may restrict Pi uptake but also enhance NO_3_^−^ absorption, suggesting that Pi contributes to nutrient balance by regulating NO_3_^−^ transporter activity. This dynamic regulatory network facilitates plant adaptability across diverse environmental conditions. In Chinese fir, despite the evolutionary disparity in the specific proteins regulating nitrogen and phosphorus nutritional balance compared to model plants, the SPX-PHR module still plays an integral role [113]. Notwithstanding, there is a notable gap in research concerning homologous genes of HHO2 within its module, hinting at potential alternative functional genes in N and P management with analogous roles. In eucalyptus, although PHR boosts Pi tolerance through PSI activation, its efficacy is significantly lower than in poplar [114]. This discussion underscores the presence of a conserved N-P co-regulation mechanism, anchored by the SPX-PHR module, across woody species.

The foundational discoveries outlined above possess considerable practical significance. Key targets, including *SPX-PHR*, *NLA*, *HHO2*, and *MYB59*, furnish an array of regulatory nodes crucial for advancing the genetics of forestry species. Utilizing molecular breeding strategies allows for the refinement of these targets’ expression patterns, facilitating the development of novel varieties characterized by optimized nutrient utilization. For instance, fine-tuning the spatial and temporal expression of *HHO2* may synergistically improve root architecture and nutrient absorption, whereas modulating *NLA* activity may bolster the stability of transport proteins.

## 5. Conclusions

In summary, our research demonstrates that, in poplar, NO_3_^−^ may stimulate the secretion of organic acids, thereby activating soil phosphorus through an upregulation of genes and activity in organic acid synthases (e.g., MDH, PEPC) and APs. This process also fine-tunes root architecture, for instance, by enhancing root length and branching through IAA/ABA mediation, which broadens the absorption interface and bolsters P activation capability, leading to a synergistic boost in P absorption efficiency. Furthermore, under conditions of low Pi availability environments, NO_3_^−^ significantly improves P absorption and recycling by co-upregulating Pi transport proteins (e.g., the PHO family), activating myo-inositol Pi metabolism (via IMP3/ITPK1 mediated PP-InsPs degradation), and enhancing hormone signaling (through IAA/SA driven P cycling). Pi triggers potential in nitrogen metabolism by activating transcription and the activity of nitrogen assimilation enzymes (GS/GOGAT/GDH). However, under low NO_3_^−^ availability environments, this leads to a metabolic imbalance characterized by high enzyme activity but low efficiency. Conversely, with adequate nitrogen, Pi fosters N absorption both physically, by enlarging the root system and its surface area, and metabolically, through improved N assimilation efficiency facilitated by increased IAA/GA accumulation and ABA signaling (e.g., *SNRK2*/*ABF*). Under conditions of low NO_3_^−^ availability environments, the supply of Pi may promote nitrogen uptake by activating the high-affinity transport system (NRT2.1/NRT3.1). Conversely, under conditions of high N, potential inhibition of *NPF2.10* could serve as negative feedback to preserve the equilibrium between N and P metabolism. The pivotal elements of nitrogen-phosphorus interaction (*SPX-PHR*, *NLA*, *HHO2*, *MYB59*) may orchestrate a dynamic network integrating transcription factor cascades, metabolic remodeling, and hormone synergy within poplar roots, thus balancing the expression and degradation of transport proteins. These insights are crucial for diminishing reliance on chemical fertilizers, mitigating forestry non-point source pollution, rejuvenating the soil nutrient reservoir, fostering soil health and biodiversity, and strengthening ecosystem resilience, thus supporting sustainable production amidst climate change. Looking ahead, by leveraging precision agronomic management (like dynamic fertilizer application) and molecular design breeding (e.g., efficient transporter engineering), we can translate the N-P interaction mechanism into environmentally friendly forestry practices, propelling a “green-efficient-resilient” forestry transformation. Nevertheless, the current research presents certain limitations.

Although it offers preliminary insights into N-P interactions in woody plants, the complex habitats of these plants and varying substrate types may impact the interaction effects. Moreover, mycorrhizal symbiosis and other biotic interactions, playing a pivotal role in natural systems, require validation in conditions mimicking field settings. Future studies should employ multi-omics approaches for comparative investigations across different woody plant ecotypes, aiming to unravel the evolutionary adaptation mechanisms underlying N-P interactions.

## Data Availability

The original contributions presented in the study are included in the article, further inquiries can be directed to the corresponding authors.

## Supplementary Materials

The following supporting information can be downloaded at: https://www.mdpi.com/article/10.3390/biology14050490/s1, Figure S1: Expression difference volcano map. (a) +N,−P vs. −N,−P, (b) −N,+P vs. −N,−P, (c) +N,+P vs. −N,+P and (d) +N,+P vs. +N,−P. Note: The abscissa is the multiple of gene expression difference between the two samples, that is, the expression of the treated sample is divided by the expression of the control sample, and the ordinate is the statistical test value of the difference in gene expression change, that is, the *p* value. The greater the −log10 (*p* value), the more significant the difference in expression, and the values of the horizontal and vertical coordinates are logarithmically processed. Each point in the figure represents a specific gene. The red point represents a significantly up-regulated gene, the green point represents a significantly down-regulated gene, and the gray point is a non-significant difference gene. After mapping all genes up, it can be learned that the point on the left is the gene with down-regulated expression difference, and the point on the right is the gene with up-regulated expression difference. The closer to the two sides and the upper point, the more significant the difference in expression; Figure S2: The GO functional enrichment of up-regulated and down-regulated DEGs under different N and P treatments was only shown in the graph showing the top 30 GOterms. (a) show the Up-regulated Gene Enrichment GO terms of the +N,−P vs. −N,−P groups and the +N,+P vs. −N,+P groups. (b) show the Down-regulated Gene Enrichment GO terms of the −N,+P vs. −N,−P groups and the +N,+P vs. +N,−P groups. (c) show the Up-regulated Gene Enrichment GO terms of the −N,+P vs. −N,−P groups and the +N,+P vs. +N,−P groups. (d) show the Down-regulated Gene Enrichment GO terms of the +N,−P vs. −N,−P groups and the +N,+P vs. −N,+P groups. Note: The vertical axis represents the Term name, the horizontal axis represents the gene set name, the size of the point represents the number of genes in this Term, and the color of the point corresponds to different *p* value ranges; Figure S3: The KEEG functional enrichment of DEGs under different N and P treatments. (a) present the +N,−P and −N,+P comparisons within the root contrast groups. (b) compare +N,−P with −N,−P and −N,+P with −N,+P in root contrast groups. Gene Ratio is defined as the proportion of enriched genes for a specific term relative to the total number of DEGs provided as input. Circle size indicates the number of DEGs, while color denotes the *p*-adjusted value; Figure S4: Families of transcription factors (TFs) under different N and P treatments and the proportion of these families. (a) show the −N,+P vs. −N,−P groups. (b) show the +N,+P vs. −N,+P groups. (c) show the +N,−P vs. −N,−P groups. (d) show the +N,+P vs. +N,−P groups; Figure S5: The correlations between fold changes of gene expression were analyzed by RNA-sequencing and RT-qPCR. RT-qPCR results were expressed on the basis of actin 2/7. The regression equation, correlation coefficient (R) and the significant level of correlation are indicated. The deep red represents a 99 % confidence interval and the light red represents a 99 % prediction interval; Figure S6: The expression heat map of TFs identified from differential genes responding to different N and P treatments; Table S1: Primers used for qRT-PCR; Table S2: Summary of Illumina sequencing reads mapped to the *P. alba* genome; Table S3: The enriched GO terms of the DEGs.

## References

[1] Heuer S., Gaxiola R., Schilling R., Herrera E.L., López A.D., Wissuwa M., Delhaize E., Rouached H. (2017). Improving phosphorus use efficiency: A complex trait with emerging opportunities. Plant J..

[2] Holford I.C.R. (1997). Soil phosphorus: Its measurement, and its uptake by plants. Soil Res..

[3] Vance C.P. (2001). Symbiotic nitrogen fixation and phosphorus acquisition. Plant nutrition in a world of declining renewable resources. Plant Physiol..

[4] Hua D., Rao R.Y., Chen W.S., Yang H., Shen Q., Lai N.W., Yang L.T., Guo J., Huang Z.R., Chen L.S. (2024). Adaptive responses of hormones to nitrogen deficiency in *Citrus sinensis* leaves and roots. Plants.

[5] Vance C.P., Uhde S.C., Allan D.L. (2003). Phosphorus acquisition and use: Critical adaptations by plants for securing a nonrenewable resource. New Phytol..

[6] Lynch J.P. (2013). Steep, cheap and deep: An ideotype to optimize water and N acquisition by maize root systems. Ann. Bot..

[7] Niu Y.F., Chai R.S., Jin G.L., Wang H., Tang C.X., Zhang Y.S. (2013). Responses of root architecture development to low phosphorus availability: A review. Ann. Bot..

[8] Song C.J., Ma K.M., Qu L.Y., Liu Y., Xu X.L., Fu B.J., Zhong J.F. (2010). Interactive effects of water, nitrogen and phosphorus on the growth, biomass partitioning and water-use efficiency of *Bauhinia faberi* seedlings. J. Arid Environ..

[9] Rubio V., Bustos R., Irigoyen M.L., Cardona L.X., Rojas T.M., Paz A.J. (2009). Plant hormones and nutrient signaling. Plant Mol. Biol..

[10] Tian Q., Chen F., Liu J., Zhang F., Mi G. (2008). Inhibition of maize root growth by high nitrate supply is correlated with reduced IAA levels in roots. J. Plant Physiol..

[11] Péret B., De Rybel B., Casimiro I., Benková E., Swarup R., Laplaze L., Beeckman T., Bennett M.J. (2009). *Arabidopsis* lateral root development: An emerging story. Trends Plant Sci..

[12] Brookbank B.P., Patel J., Gazzarrini S., Nambara E. (2021). Role of basal ABA in plant growth and development. Genes.

[13] Ondzighi-Assoume C.A., Chakraborty S., Harris J.M. (2016). Environmental nitrate stimulates abscisic acid accumulation in *Arabidopsis* root tips by releasing it from inactive stores. Plant Cell.

[14] Zhang Z., Liao H., Lucas W.J. (2014). Molecular mechanisms underlying phosphate sensing, signaling, and adaptation in plants. J. Integr. Plant Biol..

[15] Lv X., Zhang Y., Hu L., Zhang Y., Zhang B., Xia H., Du W., Fan S., Kong L. (2021). Low-nitrogen stress stimulates lateral root initiation and nitrogen assimilation in wheat: Roles of phytohormone signaling. J. Plant Growth Regul..

[16] Jiang C., Gao X., Liao L., Harberd N.P., Fu X. (2007). Phosphate starvation root architecture and anthocyanin accumulation responses are modulated by the gibberellin-DELLA signaling pathway in *Arabidopsis*. Plant Physiol..

[17] Wu Q., Jing H.K., Feng Z.H., Huang J., Shen R.F., Zhu X.F. (2022). Salicylic acid acts upstream of auxin and nitric oxide (NO) in cell wall phosphorus remobilization in phosphorus deficient rice. Rice.

[18] Khan F., Khan S., Fahad S., Faisal S., Hussain S., Ali S., Ali A. (2014). Effect of different levels of nitrogen and phosphorus on the phenology and yield of maize varieties. Am. J. Plant Sci..

[19] Maeda Y., Konishi M., Kiba T., Sakuraba Y., Sawaki N., Kurai T., Ueda Y., Sakakibara H., Yanagisawa S. (2018). A NIGT1-centred transcriptional cascade regulates nitrate signalling and incorporates phosphorus starvation signals in *Arabidopsis*. Nat. Commun..

[20] Hu B., Jiang Z., Wang W., Qiu Y., Zhang Z., Liu Y., Li A., Gao X., Liu L., Qian Y. (2019). Nitrate–NRT1.1B–SPX4 cascade integrates nitrogen and phosphorus signalling networks in plants. Nat. Plants.

[21] Medici A., Szponarski W., Dangeville P., Safi A., Dissanayake I.M., Saenchai C., Emanuel A., Rubio V., Lacombe B., Ruffel S. (2019). Identification of molecular integrators shows that nitrogen actively controls the phosphate starvation response in plants. Plant Cell.

[22] Cui Y.N., Li X.T., Yuan J.Z., Wang F.Z., Wang S.M., Ma Q. (2019). Nitrate transporter NPF7.3/NRT1.5 plays an essential role in regulating phosphate deficiency responses in *Arabidopsis*. Biochem. Biophys. Res. Commun..

[23] Bustos R., Castrillo G., Linhares F., Puga M.I., Rubio V., Pérez-Pérez J., Solano R., Leyva A., Paz-Ares J. (2010). A central regulatory system largely controls transcriptional activation and repression responses to phosphate starvation in *Arabidopsis*. PLoS Genet..

[24] Lin Y.C., Wang J., Delhomme N., Schiffthaler B., Sundström G., Zuccolo A., Nystedt B., Hvidsten T.R., de la Torre A., Cossu R.M. (2018). Functional and evolutionary genomic inferences in *Populus* through genome and population sequencing of American and European aspen. Proc. Natl. Acad. Sci. USA.

[25] Luo J., Li H., Liu T., Polle A., Peng C.H., Luo Z.B. (2013). Nitrogen metabolism of two contrasting poplar species during acclimation to limiting nitrogen availability. J. Exp. Bot..

[26] Wang C., Ying S., Huang H., Li K., Wu P., Shou H. (2009). Involvement of OsSPX1 in phosphate homeostasis in rice. Plant J..

[27] Gan H., Jiao Y., Jia J., Wang X., Li H., Shi W., Peng C., Polle A., Luo Z.B. (2016). Phosphorus and nitrogen physiology of two contrasting poplar genotypes when exposed to phosphorus and/or nitrogen starvation. Tree Physiol..

[28] Lei M., Liu Y., Zhang B., Zhao Y., Wang X., Zhou Y., Raghothama K.G., Liu D. (2011). Genetic and genomic evidence that sucrose is a global regulator of plant responses to phosphate starvation in *Arabidopsis*. Plant Physiol..

[29] Gajewska E., Niewiadomska E., Tokarz K., Słaba M., Skłodowska M. (2013). Nickel-induced changes in carbon metabolism in wheat shoots. J. Plant Physiol..

[30] Lü J., Gao X., Dong Z., Yi J., An L. (2012). Improved phosphorus acquisition by tobacco through transgenic expression of mitochondrial malate dehydrogenase from *Penicillium oxalicum*. Plant Cell Rep..

[31] Dong D., Peng X., Yan X. (2004). Organic acid exudation induced by phosphorus deficiency and/or aluminium toxicity in two contrasting soybean genotypes. Physiol. Plant..

[32] Shi W.G., Li H., Liu T.X., Polle A., Peng C.H., Luo Z.B. (2015). Exogenous abscisic acid alleviates zinc uptake and accumulation in *Populus × canescens* exposed to excess zinc. Plant Cell Environ..

[33] He J., Ma C., Ma Y., Li H., Kang J., Liu T., Polle A., Peng C., Luo Z.B. (2013). Cadmium tolerance in six poplar species. Environ. Sci. Pollut. Res..

[34] Chen S., Zhou Y., Chen Y., Gu J. (2018). fastp: An ultra-fast all-in-one FASTQ preprocessor. Bioinformatics.

[35] Kim D., Langmead B., Salzberg S.L. (2015). HISAT: A fast spliced aligner with low memory requirements. Nat. Methods.

[36] Pertea M., Pertea G.M., Antonescu C.M., Chang T.C., Mendell J.T., Salzberg S.L. (2015). StringTie enables improved reconstruction of a transcriptome from RNA-seq reads. Nat. Biotechnol..

[37] Li B., Dewey C.N. (2011). RSEM: Accurate transcript quantification from RNA-Seq data with or without a reference genome. BMC Bioinform..

[38] Love M.I., Huber W., Anders S. (2014). Moderated estimation of fold change and dispersion for RNA-seq data with DESeq2. Genome Biol..

[39] Zhu D.Y., Li Z.R., Deng S.R., Liu Q.F., Wu J.T., Chen X., Wang Y., Cheng Y., Yang L.Y., Zhou M.Y. (2023). Transcriptomic dissection underlying physiological and anatomical characteristics of poplar wood in response to changes in light intensity and nitrogen availability. Environ. Exp. Bot..

[40] Livak K.J., Schmittgen T.D. (2001). Analysis of relative gene expression data using real-time quantitative PCR and the 2^−ΔΔCT^ method. Methods.

[41] Pfaffl M.W., Horgan G.W., Dempfle L. (2002). Relative expression software tool (REST©) for group-wise comparison and statistical analysis of relative expression results in real-time PCR. Nucleic Acids Res..

[42] Xie L.L., Chen F., Zou X.L., Shen S.S., Wang X.G., Yao G.X., Xu B.B. (2019). Graphene oxide and ABA cotreatment regulates root growth of *Brassica napus* L. by regulating IAA/ABA. J. Plant Physiol..

[43] Saini S., Sharma I., Kaur N., Pati P.K. (2013). Auxin: A master regulator in plant root development. Plant Cell Rep..

[44] Ding Y., Wang Z., Mo S., Liu J., Xing Y., Wang Y., Ge C., Wang Y. (2021). Mechanism of Low Phosphorus Inducing the Main Root Lengthening of Rice. J. Plant Growth Regul..

[45] Nadira U.A., Ahmed I.M., Wu F., Zhang G. (2016). The regulation of root growth in response to phosphorus deficiency mediated by phytohormones in a Tibetan wild barley accession. Acta Physiol. Plant..

[46] Ahmad N., Jiang Z., Zhang L., Hussain I., Yang X. (2023). Insights on phytohormonal crosstalk in plant response to nitrogen stress: A focus on plant root growth and development. Int. J. Mol. Sci..

[47] Bagautdinova Z.Z., Omelyanchuk N., Tyapkin A.V., Kovrizhnykh V.V., Lavrekha V.V., Zemlyanskaya E.V. (2022). Salicylic acid in root growth and development. Int. J. Mol. Sci..

[48] Vysotskaya L., Akhiyarova G., Feoktistova A., Akhtyamova Z., Korobova A., Ivanov I., Dodd I., Kuluev B., Kudoyarova G. (2020). Effects of phosphate shortage on root growth and hormone content of barley depend on capacity of the roots to accumulate ABA. Plants.

[49] Roychoudhry S., Kepinski S. (2022). Auxin in root development. Cold Spring Harb. Perspect. Biol..

[50] Ma W.Y., Li J.J., Qu B.Y., He X., Zhao X.Q., Li B., Fu X.D., Tong Y.P. (2014). Auxin biosynthetic gene 2 is involved in low nitrogen-mediated reprogramming of root architecture in *Arabidopsis*. Plant J..

[51] Shao A., Ma W., Zhao X., Hu M., He X., Teng W., Li H., Tong Y. (2017). The Auxin Biosynthetic TRYPTOPHAN AMINOTRANSFERASE RELATED TaTAR2.1-3A Increases Grain Yield of Wheat. Plant Physiol..

[52] Jia Z., Giehl R.F.H., von Wirén N. (2021). Local auxin biosynthesis acts downstream of brassinosteroids to trigger root foraging for nitrogen. Nat. Commun..

[53] Yang R., Wang S., Zou H., Li L., Li Y., Wang D., Xu H., Cao X. (2021). R2R3-MYB transcription factor *Sm*MYB52 positively regulates biosynthesis of salvianolic acid B and inhibits root growth in *Salvia miltiorrhiza*. Int. J. Mol. Sci..

[54] Wild M., Davière J.M., Cheminant S., Regnault T., Baumberger N., Heintz D., Baltz R., Genschik P., Achard P. (2012). The *Arabidopsis* DELLA RGA-LIKE3 is a direct target of MYC2 and modulates jasmonate signaling responses. Plant Cell.

[55] Kurita Y., Baba K., Ohnishi M., Matsubara R., Kosuge K., Anegawa A., Shichijo C., Ishizaki K., Kaneko Y., Hayashi M. (2017). Inositol hexakis phosphate is the seasonal phosphorus reservoir in the deciduous woody plant *Populus alba* L.. Plant Cell Physiol..

[56] Luan J., Xin M., Qin Z. (2023). Genome-wide identification and functional analysis of the roles of *SAUR* gene family members in the promotion of cucumber root expansion. Int. J. Mol. Sci..

[57] Spartz A.K., Lor V.S., Ren H., Olszewski N.E., Miller N.D., Wu G., Spalding E.P., Gray W.M. (2017). Constitutive expression of *Arabidopsis SMALL AUXIN UP RNA19* (*SAUR19*) in tomato confers auxin-independent hypocotyl elongation. Plant Physiol..

[58] Zhao Y., Xing L., Wang X., Hou Y.J., Gao J., Wang P., Duan C.G., Zhu X., Zhu J.K. (2014). The ABA receptor PYL8 promotes lateral root growth by enhancing MYB77-dependent transcription of auxin-responsive genes. Sci. Signal..

[59] Wang J., Li Y., Hu Y., Zhu S. (2024). Jasmonate induces translation of the *Arabidopsis* transfer RNA-binding protein YUELAO1, which activates MYC2 in jasmonate signaling. Plant Cell.

[60] Devaiah B.N., Madhuvanthi R., Karthikeyan A.S., Raghothama K.G. (2009). Phosphate starvation responses and gibberellic acid biosynthesis are regulated by the MYB62 transcription factor in *Arabidopsis*. Mol. Plant.

[61] Hussain S.J., Khan N.A., Anjum N.A., Masood A., Khan M.I.R. (2021). Mechanistic elucidation of salicylic acid and sulphur-induced defence systems, nitrogen metabolism, photosynthetic, and growth potential of mungbean (*Vigna radiata*) under salt stress. J. Plant Growth Regul..

[62] Xu G., Fan X., Miller A. (2012). Plant nitrogen assimilation and use efficiency. Annu. Rev. Plant Biol..

[63] Li H., Li M., Luo J., Cao X., Qu L., Gai Y., Jiang X., Liu T., Bai H., Janz D. (2012). N-fertilization has different effects on the growth, carbon and nitrogen physiology, and wood properties of slow- and fast-growing *Populus* species. J. Exp. Bot..

[64] Chen Y.H., Nguyen T.H.N., Qin J.J., Jiao Y., Li Z.L., Ding S., Lu Y., Liu Q.F., Luo Z.B. (2018). Phosphorus assimilation of Chinese fir from two provenances during acclimation to changing phosphorus availability. Environ. Exp. Bot..

[65] Gan H.H., Chu J.M., Shi W.G., Luo Z.B. (2024). Physiological and transcriptomic regulation of *Populus simonii* fine roots exposed to a heterogeneous phosphorus environment in soil. Environ. Exp. Bot..

[66] Bakrim N., Nhiri M., Pierre J.N., Vidal J. (1998). Metabolite control of *Sorghum* C4 phosphoenolpyruvate carboxylase catalytic activity and phosphorylation state. Photosynth. Res..

[67] Ha S., Tran L.S. (2014). Understanding plant responses to phosphorus starvation for improvement of plant tolerance to phosphorus deficiency by biotechnological approaches. Crit. Rev. Biotechnol..

[68] Shane M.W., Fedosejevs E.T., Plaxton W.C. (2013). Reciprocal control of anaplerotic phosphoenolpyruvate carboxylase by in vivo monoubiquitination and phosphorylation in developing proteoid roots of phosphate-deficient harsh hakea. Plant Physiol..

[69] Tian W.H., Ye J.Y., Cui M.Q., Chang J.B., Liu Y., Li G.X., Wu Y.R., Xu J.M., Harberd N.P., Mao C.Z. (2021). A transcription factor STOP1-centered pathway coordinates ammonium and phosphate acquisition in *Arabidopsis*. Mol. Plant.

[70] Chiariello N.R., Mooney H.A., Williams K., Pearcy R.W., Ehleringer J.R., Mooney H.A., Rundel P.W. (1989). Growth, carbon allocation and cost of plant tissues. Plant Physiological Ecology: Field Methods and Instrumentation.

[71] Shipley B., Peters R.H. (1990). A test of the tilman model of plant strategies: Relative growth rate and biomass partitioning. Am. Nat..

[72] Umaña M.N., Cao M., Lin L.X., Swenson N.G., Zhang C.C. (2021). Trade-offs in above- and below-ground biomass allocation influencing seedling growth in a tropical forest. J. Ecol..

[73] Guilbeault-Mayers X., Turner B.L., Laliberté E. (2020). Greater root phosphatase activity of tropical trees at low phosphorus despite strong variation among species. Ecology.

[74] Lambers H., Finnegan P.M., Jost R., Plaxton W.C., Shane M.W., Stitt M. (2015). Phosphorus nutrition in proteaceae and beyond. Nat. Plants.

[75] Duan X.J., Jin K., Ding G.D., Wang C., Cai H.M., Wang S.L., White P.J., Xu F.S., Shi L. (2020). The impact of different morphological and biochemical root traits on phosphorus acquisition and seed yield of *Brassica napus*. Field Crops Res..

[76] Zhu S., Luo L., Zhang X., Zhao M., Wang X., Zhang J., Wan Q., Li X., Wan Y., Zhang K. (2022). Study on the relationship of root morphology and phosphorus absorption efficiency with phosphorus uptake capacity in 235 Peanut (*Arachis hypogaea* L.) germplasms. Front. Environ. Sci..

[77] Li X.X., Zeng R.S., Liao H. (2016). Improving crop nutrient efficiency through root architecture modifications. J. Integr. Plant Biol..

[78] Levi M., Gratton E., Forster I.C., Hernando N., Wagner C.A., Biber J., Sorribas V., Murer H. (2019). Mechanisms of phosphate transport. Nat. Rev. Nephrol..

[79] Pfaff J., Denton A.K., Usadel B., Pfaff C. (2020). Phosphate starvation causes different stress responses in the lipid metabolism of tomato leaves and roots. Biochim. Biophys. Acta (BBA)—Mol. Cell Biol. Lipids.

[80] Jost R., Pharmawati M., Lapis G.H.R., Rossig C., Berkowitz O., Lambers H., Finnegan P.M. (2015). Differentiating phosphate-dependent and phosphate-independent systemic phosphate-starvation response networks in *Arabidopsis thaliana* through the application of phosphite. J. Exp. Bot..

[81] Guan Z., Zhang Q., Zhang Z., Zuo J., Chen J., Liu R., Savarin J., Broger L., Cheng P., Wang Q. (2022). Mechanistic insights into the regulation of plant phosphate homeostasis by the rice SPX2-PHR2 complex. Nat. Commun..

[82] Dong J., Ma G., Sui L., Wei M., Satheesh V., Zhang R., Ge S., Li J., Zhang T.E., Wittwer C. (2019). Inositol pyrophosphate InsP_8_ acts as an intracellular phosphate signal in *Arabidopsis*. Mol. Plant.

[83] Zhou J., Hu Q.L., Xiao X.L., Yao D.Q., Ge S.H., Ye J., Li H.J., Cai R.J., Liu R.Y., Meng F.G. (2021). Mechanism of phosphate sensing and signaling revealed by rice SPX1-PHR2 complex structure. Nat. Commun..

[84] Park S.H., Jeong J.S., Huang C.H., Park B.S., Chua N.H. (2023). Inositol polyphosphates-regulated polyubiquitination of PHR1 by NLA E3 ligase during phosphate starvation response in *Arabidopsis*. New Phytol..

[85] Laha D., Parvin N., Hofer A., Giehl R.F.H., Fernandez-Rebollo N., von Wirén N., Saiardi A., Jessen H.J., Schaaf G. (2019). Arabidopsis ITPK1 and ITPK2 have an evolutionarily conserved phytic acid kinase activity. ACS Chem. Biol..

[86] Riemer E., Qiu D., Laha D., Harmel R.K., Gaugler P., Gaugler V., Frei M., Hajirezaei M.-R., Laha N.P., Krusenbaum L. (2021). ITPK1 is an InsP_6_/ADP phosphotransferase that controls phosphate signaling in *Arabidopsis*. Mol. Plant.

[87] Paul P., Sharma S., Pandey R. (2023). Phosphorus scavenging and remobilization from root cell walls under combined nitrogen and phosphorus stress is regulated by phytohormones and nitric oxide cross-talk in wheat. J. Plant Growth Regul..

[88] Lezhneva L., Kiba T., Feria B.A.B., Lafouge F., Boutet M.S., Zoufan P., Sakakibara H., Daniel V.F., Krapp A. (2014). The *Arabidopsis* nitrate transporter NRT2.5 plays a role in nitrate acquisition and remobilization in nitrogen-starved plants. Plant J..

[89] Jia L., Hu D., Wang J., Liang Y., Li F., Wang Y., Han Y. (2023). Genome-wide identification and functional analysis of nitrate transporter genes (*NPF*, *NRT2* and *NRT3*) in maize. Int. J. Mol. Sci..

[90] Jeschke W.D., Kirkby E.A., Peuke A.D., Pate J.S., Hartung W. (1997). Effects of P deficiency on assimilation and transport of nitrate and phosphate in intact plants of castor bean (*Ricinus communis* L.). J. Exp. Bot..

[91] Siddiqi M.Y., Glass A.D.M., Ruth T.J., Rufty T.W. (1990). Studies of the uptake of nitrate in barley: I. Kinetics of ^13^NO^3−^ Influx. Crop Sci..

[92] Xing J., Cao X., Zhang M., Wei X., Zhang J., Wan X. (2023). Plant nitrogen availability and crosstalk with phytohormones signallings and their biotechnology breeding application in crops. Plant Biotechnol. J..

[93] David L.C., Berquin P., Kanno Y., Seo M., Daniel V.F., Ferrario M.S. (2016). N availability modulates the role of NPF3.1, a gibberellin transporter, in GA-mediated phenotypes in *Arabidopsis*. Planta.

[94] Wang Z., Ruan W., Shi J., Zhang L., Xiang D., Yang C., Li C., Wu Z., Liu Y., Yu Y. (2014). Rice *SPX1* and *SPX2* inhibit phosphate starvation responses through interacting with *PHR2* in a phosphate-dependent manner. Proc. Natl. Acad. Sci. USA.

[95] Shi J., Hu H., Zhang K., Zhang W., Yu Y., Wu Z., Wu P. (2014). The paralogous SPX3 and SPX5 genes redundantly modulate Pi homeostasis in rice. J. Exp. Bot..

[96] Sun L., Song L., Zhang Y., Zheng Z., Liu D. (2016). *Arabidopsis* PHL2 and PHR1 act redundantly as the key components of the central regulatory system controlling transcriptional responses to phosphate starvation. Plant Physiol..

[97] Wu Y., Chen C., Wang G. (2024). Inoculation with arbuscular mycorrhizal fungi improves plant biomass and nitrogen and phosphorus nutrients: A meta-analysis. BMC Plant Biol..

[98] He K., Du J., Han X., Li H., Kui M., Zhang J., Huang Z., Fu Q., Jiang Y., Hu Y. (2023). PHOSPHATE STARVATION RESPONSE1 (PHR1) interacts with JASMONATE ZIM-DOMAIN (JAZ) and MYC2 to modulate phosphate deficiency-induced jasmonate signaling in *Arabidopsis*. Plant Cell.

[99] Ribot C., Wang Y., Poirier Y. (2008). Expression analyses of three members of the *AtPHO1* family reveal differential interactions between signaling pathways involved in phosphate deficiency and the responses to auxin, cytokinin, and abscisic acid. Planta.

[100] Shin H., Shin H.S., Chen R., Harrison M.J. (2006). Loss of At*4* function impacts phosphate distribution between the roots and the shoots during phosphate starvation. Plant J..

[101] Huang T.K., Han C.L., Lin S.I., Chen Y.J., Tsai Y.C., Chen Y.R., Chen J.W., Lin W.Y., Chen P.M., Liu T.Y. (2013). Identification of downstream components of ubiquitin-conjugating enzyme PHOSPHATE2 by quantitative membrane proteomics in *Arabidopsis* roots. Plant Cell.

[102] Lin W.Y., Huang T.K., Chiou T.J. (2013). Nitrogen limitation adaptation, a target of microRNA827, mediates degradation of plasma membrane-localized phosphate transporters to maintain phosphate homeostasis in *Arabidopsis*. Plant Cell.

[103] Liu W.W., Sun Q., Wang K., Du Q.G., Li W.X. (2017). Nitrogen limitation adaptation (NLA) is involved in source-to-sink remobilization of nitrate by mediating the degradation of NRT1.7 in *Arabidopsis*. New Phytol..

[104] Durand M., Brehaut V., Clement G., Kelemen Z., Macé J., Feil R., Duville G., Launay A.A., Roux C.P.L., Lunn J.E. (2023). The *Arabidopsis* transcription factor NLP2 regulates early nitrate responses and integrates nitrate assimilation with energy and carbon skeleton supply. Plant Cell.

[105] Zhang Z., Li Z., Wang W., Jiang Z., Guo L., Wang X., Qian Y., Huang X., Liu Y., Liu X. (2021). Modulation of nitrate-induced phosphate response by the MYB transcription factor RLI1/HINGE1 in the nucleus. Mol. Plant.

[106] Du X.Q., Wang F.L., Li H., Jing S., Yu M., Li J., Wu W.H., Kudla J., Wang Y. (2019). The transcription factor MYB59 Regulates K^+^/NO_3_^−^ translocation in the *Arabidopsis* Response To Low K^+^ stress. Plant Cell.

[107] Nagarajan V.K., Satheesh V., Poling M.D., Raghothama K.G., Jain A. (2016). *Arabidopsis* MYB-related HHO_2_ exerts a regulatory influence on a subset of root traits and genes governing phosphate homeostasis. Plant Cell Physiol..

[108] Ueda Y., Yanagisawa S. (2023). Transcription factor module NLP–NIGT1 fine-tunes *NITRATE TRANSPORTER2.1* expression. Plant Physiol..

[109] Li Q., Zhou L., Li Y., Zhang D., Gao Y. (2021). Plant NIGT1/HRS1/HHO transcription factors: Key regulators with multiple roles in plant growth, development, and stress responses. Int. J. Mol. Sci..

[110] Wang X., Wang H.F., Chen Y., Sun M.M., Wang Y., Chen Y.F. (2020). The transcription factor NIGT1.2 modulates both phosphate uptake and nitrate influx during phosphate starvation in *Arabidopsis* and *Maize*. Plant Cell.

[111] Zhuo M.N., Sakuraba Y., Yanagisawa S.C. (2024). Dof1.7 and NIGT1 transcription factors mediate multilayered transcriptional regulation for different expression patterns of *NITRATE TRANSPORTER2* genes under nitrogen deficiency stress. New Phytol..

[112] Zhang Y.X., Zhang Q.Q., Guo M.N., Wang X.Q., Li T.J., Wu Q.Y., Li L.H., Yi K.K., Ruan W.Y. (2023). NIGT1 represses plant growth and mitigates phosphate starvation signaling to balance the growth response tradeoff in rice. J. Integr. Plant Biol..

[113] Xu H.M., Deng L.C., Zhou X., Xing Y.F., Li G.L., Chen Y., Huang Y., Ma X.Q., Liu Z.J., Li M. (2024). Unveiling the PHR-centered regulatory network orchestrating the phosphate starvation signaling in Chinese fir (*Cunninghamia lanceolata*). bioRxiv.

[114] Bulgarelli R.G., Araujo P., Engel E., Mazzafera P., de Andrade S.A.L. (2024). Eucalypt seedlings lack a clear phosphate starvation response under low phosphorus availability. Theor. Exp. Plant Physiol..

