# Supplementary material for: The Impact of Nitrogen and Phosphorus Interaction on Growth, Nutrient Absorption, and Signal Regulation in Woody Plants
Source: Biology (Basel). 2025 Apr 30;14(5):490. doi: 10.3390/biology14050490 (PMC12108901; doi:10.3390/biology14050490)
Supplement: Supplementary file 1 [file biology-14-00490-s001.zip › figures.pdf]

Supplementary materials

The Impact of Nitrogen and Phosphorus Interaction on Growth, Nutrient Absorption, and Signal Regulation in Woody Plants

Xiaan Tang, Yi Zhang, Panpan Meng, Yingke Yuan, Changhao Li, Xiaotan Zhi, Chunyan Wang

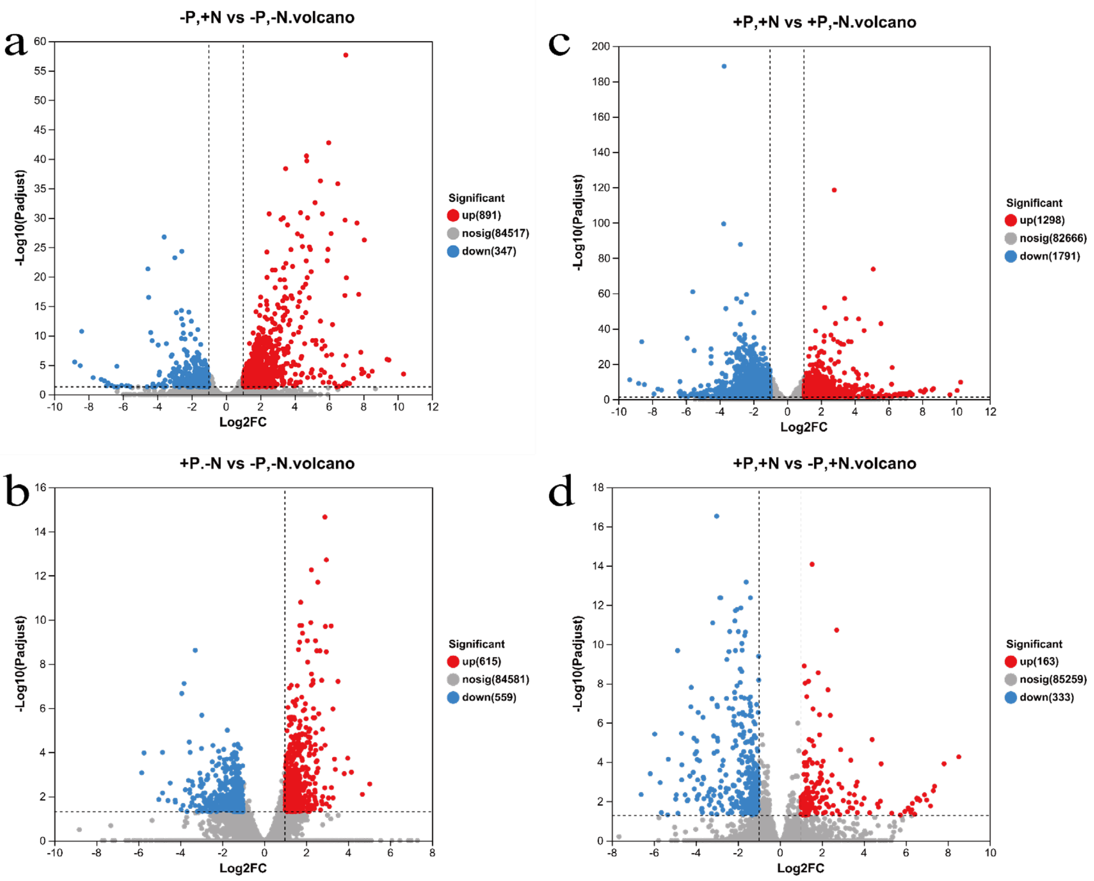

Supplementary Figure S1

Expression difference volcano map. (a) +N,-P vs. -N,-P, (b) -N,+P vs. -N,-P, (c) +N,+P vs. -N,+P and (d) +N,+P vs. +N,-P. Note: The abscissa is the multiple of gene expression difference between the two samples, that is, the expression of the treated sample is divided by the expression of the control sample, and the ordinate is the statistical test value of the difference in gene expression change, that is, the *p* value. The greater the -log10 (*p* value), the more significant the difference in expression, and the values of the horizontal and vertical coordinates are logarithmically processed. Each point in the figure represents a specific gene. The red point represents a significantly up-regulated gene, the green point represents a significantly down-regulated gene, and the gray point is a non-significant difference gene. After mapping all genes up, it can be learned that the point on the left is the gene with down-regulated expression difference, and the point on the right is the gene with up-regulated expression difference. The closer to the two sides and the upper point, the more significant the difference in expression.

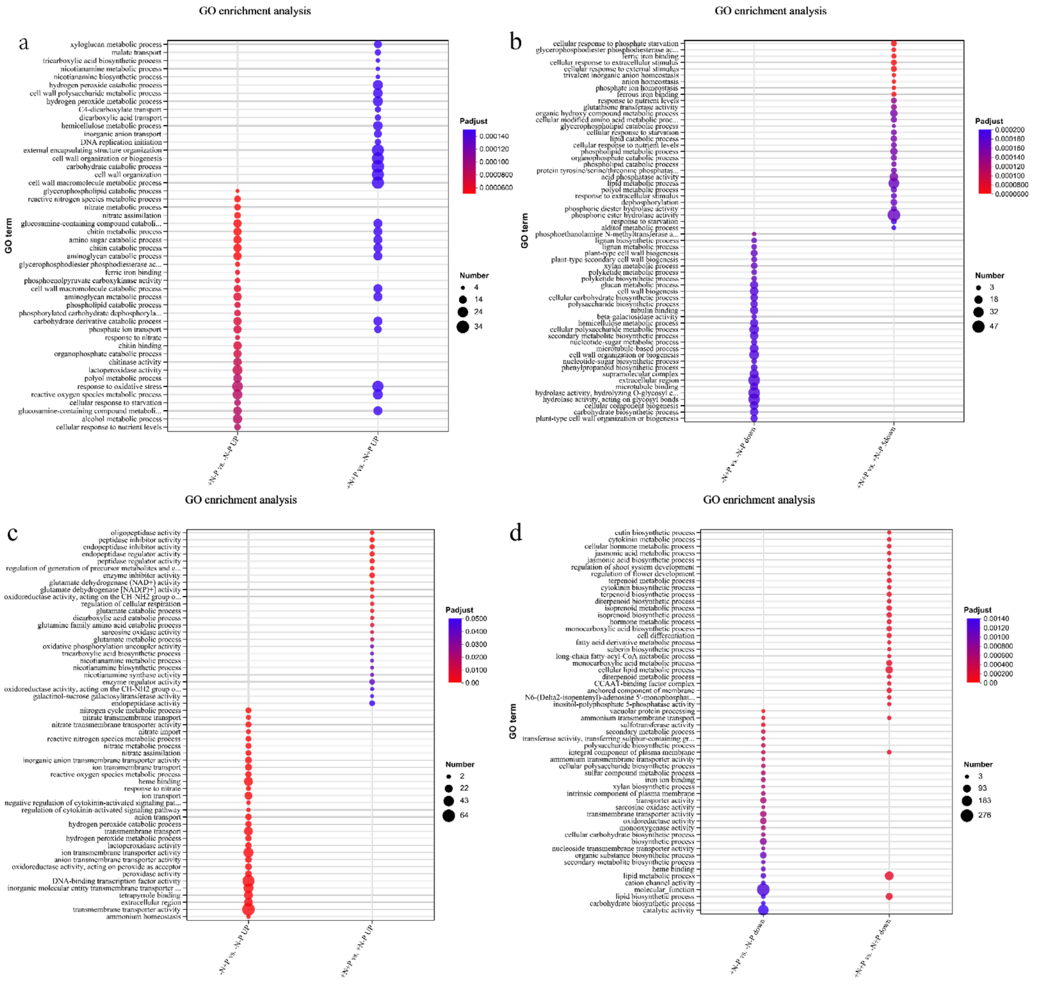

Supplementary Figure S2 The GO functional enrichment of up-regulated and down-regulated DEGs under different N and P treatments was only shown in the graph showing the top 30 GOterms. (a) show the Up-regulated Gene Enrichment GO terms of the +N,-P vs. -N,-P groups and the +N,+P vs. -N,+P groups. (b) show the Down-regulated Gene Enrichment GO terms of the -N,+P vs. -N,-P groups and the +N,+P vs. +N,-P groups. (c) show the Up-regulated Gene Enrichment GO terms of the -N,+P vs. -N,-P groups and the +N,+P vs. +N,-P groups. (d) show the Down-regulated Gene Enrichment GO terms of the +N,-P vs. -N,-P groups and the +N,+P vs. -N,+P groups. Note: The vertical axis represents the Term name, the horizontal axis represents the gene set name, the size of the point represents the number of genes in this Term, and the color of the point corresponds to different *P* value ranges.

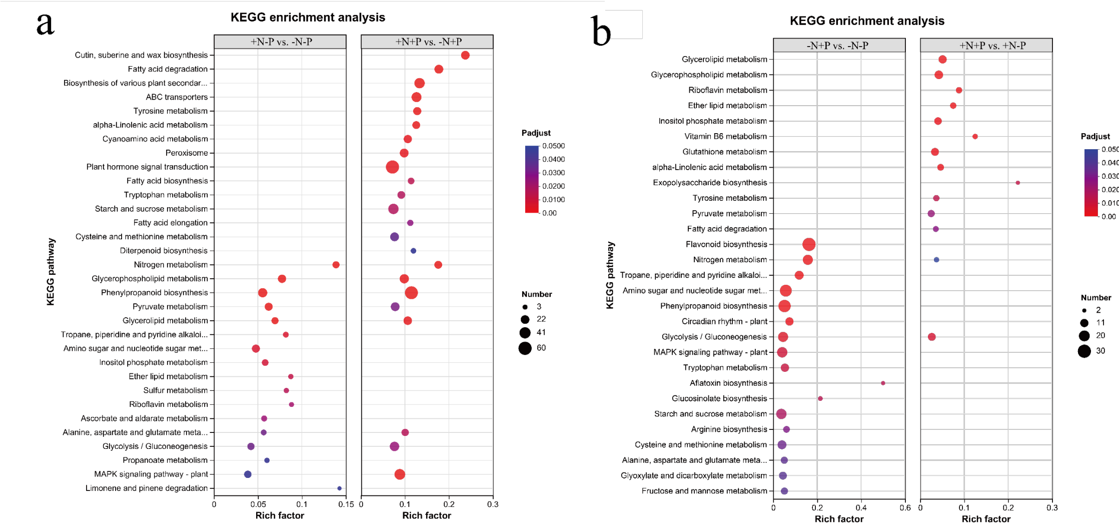

Supplementary Figure S3 The KEEG functional enrichment of DEGs under different N and P treatments. (a) present the +N,-P and -N,+P comparisons within the root contrast groups. (b) compare +N,-P with -N,-P and -N,+P with -N,+P in root contrast groups. Gene Ratio is defined as the proportion of enriched genes for a specific term relative to the total number of DEGs provided as input. Circle size indicates the number of DEGs, while color denotes the *p*-adjusted value.

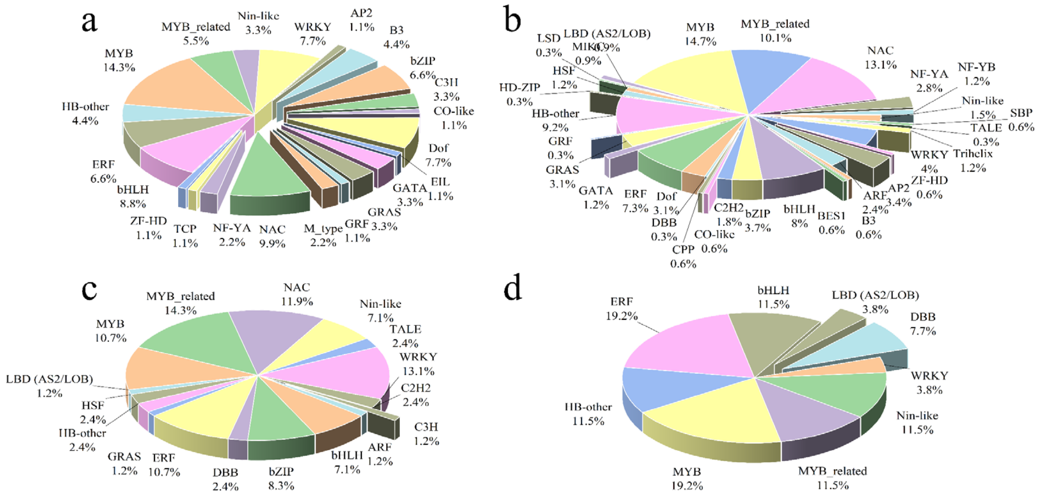

Supplementary Figure S4. Families of transcription factors (TFs) under different N and P treatments and the proportion of these families. (a) show the -N,+P vs. -N,-P groups. (b) show the +N,+P vs. -N,+P groups. (c) show the +N,-P vs. -N,-P groups. (d) show the +N,+P vs. +N,-P groups.

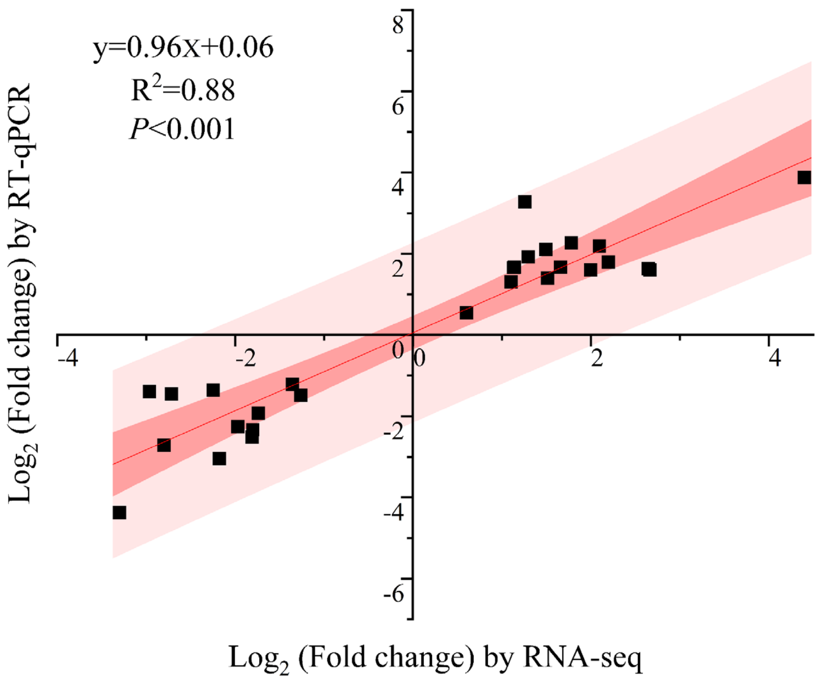

Supplementary Figure S5 The correlations between fold changes of gene expression were analyzed by RNA-sequencing and RT-qPCR. RT-qPCR results were expressed on the basis of *actin 2/7*. The regression equation, correlation coefficient (R) and the significant level of correlation are indicated. The deep red represents a 99 % confidence interval and the light red represents a 99 % prediction interval.

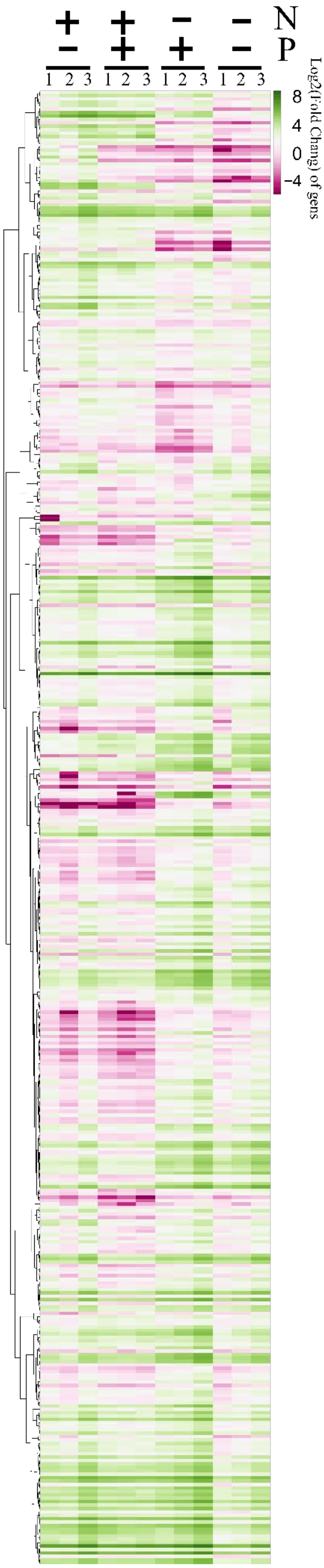

Supplementary Figure S6 The expression heat map of TFs identified from differential genes responding to different N and P treatments.
